# Modeling the Spread of Multiple Concurrent Contagions on Networks

**DOI:** 10.1371/journal.pone.0095669

**Published:** 2014-06-12

**Authors:** Angel Stanoev, Daniel Trpevski, Ljupco Kocarev

**Affiliations:** Macedonian Academy of Sciences and Arts, Skopje, Macedonia; Inserm & Universite Pierre et Marie Curie, France

## Abstract

Many contagions spread over various types of communication networks and their spreading dynamics have been extensively studied in the literature. Here we propose a general model for the concurrent spread of an arbitrary number of contagions in complex networks. The model is stochastic and runs in discrete time, and includes two widely used mechanisms by which a node can change its state. The first, termed the spontaneous state change mechanism, describes spontaneous transition to another state, while the second, termed the contact-induced state change mechanism, describes acquiring other contagions due to contact with the neighbors. We consider reactive discrete-time spreading processes of multiple concurrent contagions where time steps are of finite size without neglecting the possibility of multiple infecting events in a single time step. An essential element for making the model numerically tractable is the use of an approximation for the probability that a node transits to a specific state given any set of neighboring states. Different transmission probabilities may be present between each pair of states. We also derive corresponding continuous–time equations that are simple and intuitive. The model includes many well-known epidemic and rumor spreading models as a special case and it naturally captures spreading processes in multiplex networks.

## Introduction

Epidemiological models, developed as tools for analyzing the spread and control of infectious diseases, have also been adapted across many scientific fields such as ecology, immunology, social science, computer science, marketing and economy. They focus on modeling the dynamics of contagious entities (also called “memes” in the literature) as diverse as communicable diseases, cultural characteristics (such as religious beliefs, fads or innovations), addictions, or information spread (through rumors, e-mail messages, web blogs, peer-to-peer computer networks, etc). Both deterministic and stochastic epidemic models have been suggested, addressing complementary questions [1]–[4]. Some well-known classical models are deterministic, and include, for example, the SIR (susceptible–infected–recovered) differential equation model of Kermack and McKendrick, which has proven useful in ascertaining gross factors affecting the rate of growth and the final size of an epidemic [5]. Stochastic models are preferable when studying a small community where the contact structure in the community contains small complete graphs, with households and other local social networks being common examples. But even when considering large communities, at which deterministic models primarily aim, some additional questions have been raised that can only be addressed with stochastic models [4]. Deterministic counterparts, working with the expected values of the corresponding stochastic models, are proposed for many of them to answer one of the fundamental questions for the propagation of a single contagion: will it infect a significant portion of the network or will it die out fast? Specifically, the existence of threshold values for the model parameters over which epidemic proportions can be reached has been studied. Earlier approaches have used a mean-field approximation [6], assuming homogeneous environments which are suited for simple network topologies. Afterwards, a heterogeneous mean-field (HMF) approximation has been introduced, which assumes that nodes with same degree behave in the same manner [7], [8]. This approach can be applied to power-law networks, but its main assumption is not empirically or phenomenologically justified and it can result in different levels of accuracy [9]. One of the recent approaches, the so-called nonlinear dynamical system (NLDS) approach, describing the evolution of the probabilities of infection for every node [10]–[14] is widely accepted. As the number of states in the Markov chain which describes the dynamics of the whole network grows exponentially with the number of nodes, independence between the marginal distributions of the nodes is assumed in order to reduce the complexity of the models. This turns out to be a valid assumption in the vast majority of complex networks because the inherent topological disorder makes dynamical correlations not persistent.

Regarding the spread of more than one contagious entity, both deterministic as well as several stochastic models have been suggested [15]–[28]. However, the spreading rules in these models are specific to the problems the models are addressing. In [24] a SIR-like consecutive spreading of multiple viruses on special random networks has been introduced. It has afterwards been adapted for concurrent spreading of multiple viruses, and percolation analysis, which is suitable for SIR-like models, has been used to predict the epidemic sizes [25]. A generalization of the SIR model for two infections, the 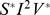 model, is proposed in [20], where a node's state is classified in one of the three categories: susceptible, infected or recovered (vigilant/vaccinated). The 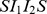 model is introduced in [20], where a susceptible node can become infected with or recover from one of two infections, and an extension is later made where it is possible that a node possesses both infections at the same time [19]. Spreading of multiple contagions has also been studied in the context of multiplex networks. Such examples are discussed in [27], [28] and [29], where the interplay between two different SIS propagations on two distinct layers of the multiplex network is considered.

Nonetheless, modeling the spread of multiple concurrent contagions with a discrete-time reactive process presents a significant distinct problem, and that is the question of how to deal with multiple simultaneous infecting events by different contagions in a single time step. Most of the proposed methods avoid this problem by assuming infinitesimally small time step sizes or asynchronous infections, allowing only a single event in a given time step. We are not aware of any work that studies the interplay between multiple competing contagions in networks and addresses the aforementioned problem directly, without neglecting the possibility of multiple simultaneous infecting events. The purpose of this paper is to propose such a model. Regarding the case with infinitesimally small time steps, we show that the differential equations of our model, derived from the discrete-time equations, naturally become simplified due to the lack of strong competition between the contagions on the level of a single node. Also, our work is among the first to propose a discrete-time stochastic model for the spread of an arbitrary, but finite, number of contagions which is general enough to describe a large class of spreading processes. The model we propose has two mechanisms of transition between two states of a node. The contact-induced transition mechanism is infection with other contagions due to contact with the neighbors, while the spontaneous transition mechanism characterizes a spontaneous transition to another model state without any contact with the neighbors. These two mechanisms encompass what is commonly met in the literature of modeling spreading processes and, as a result, our model generalizes some well-known single- and multiple-contagion spreading models.

As Daley and Kendall have pointed out, a mathematical model for the spreading of contagious entities can be (and has been) constructed in a number of different ways, depending on the mechanism postulated to describe the growth and decay of the actual spreading process [30]. Here we briefly describe two constructions commonly met in the literature. Consider a finite closed population of entities (agents) which is divided into three mutually exclusive and exhaustive classes 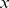, 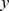, 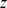. If we assume that the only two transitions allowed are: from (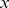, 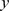, 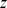) to (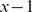, 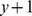, 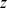) at a rate proportional to 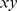, and from (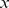,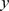, 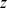) to (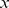, 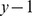, 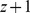) at a rate proportional to 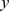, then we obtain the deterministic Kermack-McKendrick epidemic model or stochastic SIR model, depending on what 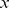, 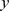, 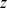 are. On the other hand, assuming a transition from (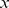, 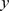, 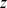) to (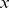, 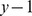, 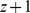) at a rate proportional to 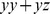, one obtains the deterministic Daley-Kendall or Maki-Thompson model of rumor spreading. In the first construction, as is indicated by the transition rate 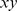, the growth in the number of entities is in one of the classes involved in the interaction. This particular interaction is between infected and susceptible individuals, and as a result, the number of infected individuals grows. In the second construction the increase in the number of entities in class 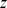 is additionally a result of interaction between classes unrelated to 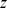, as is given with 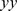 in the transition rate. In the Daley-Kendall and Maki-Thompson models this is based on the plausible hypothesis that an active spreader stops telling the rumor because when contacting another spreader it learns that the rumor has lost its news value. They both represent examples of the contact-induced transition mechanism. The spontaneous transition mechanism is also present in the first construction, where the transition rate from class 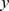 to 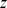 is proportional only to the number of members in class 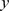.

The model suggested in this paper has three main characteristics. Firstly, it belongs to the class of stochastic discrete-time models, applies to arbitrary graphs, and can quantify the microscopic dynamics at the individual level by computing the probability that any given node is in a given state. A key instrument which we use to make this general model applicable for simulations is an approximation for the exact probability that a node will adopt a specific state from its neighbors. The approximation overcomes the challenge presented by the possibility of multiple simultaneous infections from the neighbors in a given time step which is a consequence of the finite sizes of the time steps.

Secondly, from the model one can derive its deterministic counterpart, both in difference and in differential equation form. Indeed, by assuming that the states of each node are independent random variables, one can derive a system of probability equations, which, in fact, represents a deterministic nonlinear dynamical system. Further, using a homogeneous or heterogeneous mean-field approximation for these deterministic dynamical systems, one can obtain models of differential equations describing the macroscopic spreading phenomena.

Thirdly, the model generalizes the stochastic SIR, SIS and SIRS models for an arbitrary number of contagions or states and also suggests a stochastic microscopic Markov chain version of the deterministic Maki-Thompson model for an arbitrary number of rumors. It is general enough to capture spreading in multiplex networks as well.

We stress that although it is apparent that some, or even all of the real-world spreading phenomena do not fit into general simplified schemes and require special consideration of their details as they have characteristic modes of transmission, we believe that studying simple models may nevertheless be useful for understanding underlying principles of the spreading processes. At last, for the purpose of term unification, we shall refer to the contagious entities whose spread we model as infections or contagions, by analogy with the epidemiology literature, while also bearing in mind the generality of the model to describe the spreading of any other kind of entities through a network. We will often refer to them as states as well, from the representation of the dynamics of each node as a Markov chain.

## The Model

### Single-contagion spreading models

Before we define the model, we briefly turn our attention to the susceptible-infected-susceptible (SIS) model which we use as a paradigmatic model for the spread of *one* infection in a population of individuals connected in an arbitrary topology. The network of connections is represented by a simple, undirected and connected graph of 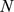 nodes whose adjacency matrix 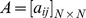 is a binary valued matrix stating whether nodes 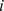 and 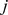 are connected (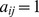) or not (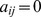). An individual, which is represented by a node in the network, can be in either a susceptible (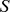) or infected (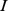) state. A susceptible node is healthy and it can receive the infection from infected neighbors. An infected node transmits the infection with probability 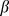 when contacting a neighbor. A successful transmission to a susceptible node causes it to become infected, and a successful transmission to an infected node has no effect. The probability that an infected node is cured and reverts back to the susceptible state is 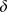.

Depending on the number of contacts a node makes, several spreading processes have been studied [12]. The one most often to be found in the literature is the reactive process. In this process an infected node contacts all of its neighbors and attempts to transmit the infection to each of them with probability 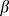. Hence, a susceptible node can receive the infection from more than one neighbor. In this case, it chooses one of the successful transmissions and adopts the infection transmitted by that contact. Since the SIS model describes the spread of only one infection in the network, it is irrelevant which particular successful contact a node chooses in order to adopt the infection.

A discrete-time stochastic mathematical model of the thus described process is as follows. The state of node 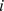 at time 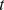 is described by a state vector containing a 1 in the component corresponding to the current state of the node, and 0 in the other:
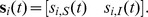



The probability mass function which states the probability of being in each of the states is given with the probability vector
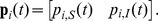



**Figure 1:**
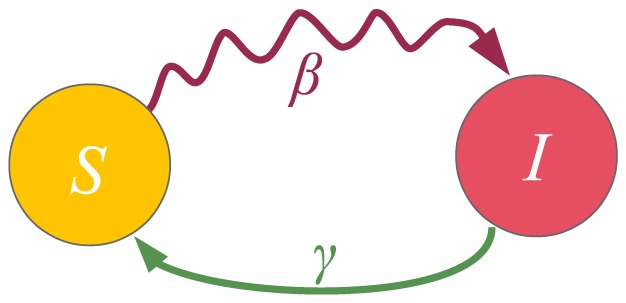
State diagram for the SIS model. The diagram shows the dynamics of a single node. A susceptible node can become infected by contacting its infected neighbors, with probability 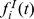. On the other hand, infected nodes spontaneously recover with probability 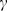, and become susceptible again. The contact-induced transition mechanism is represented by a curvy arrow, whereas the spontaneous transition mechanism is represented by a less curvy arrow.

The model equations describing the time evolution of the probability vector for each node are:
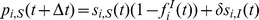


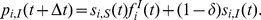
where 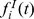 is the probability that node 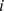 receives the infection from at least one infected neighbor. When writing an expression for 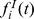, it is commonly assumed that transmission events in the current or past time steps are independent of each other. The specific spreading process determines the form of 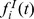 and for the reactive process it reads



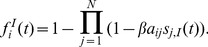
(1)The product in (1) is the probability of the event that none of the infected neighbors (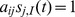) transmits the infection to node 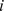. Hence, 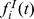 is in fact given with the probability of the opposite event, which is that at least one infected neighbor manages to transmit the infection to node 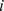. The state diagram that summarizes the Markov chain diagram for the dynamics of a single node in the SIS model is given in Fig. 1.

In order to infer the analogy of this (reactive) spreading process where a single contagious entity spreads in a network to the one where more contagious entities spread simultaneously, we use the binomial theorem to rewrite (1) for a node 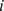 with 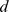 infected neighbors:
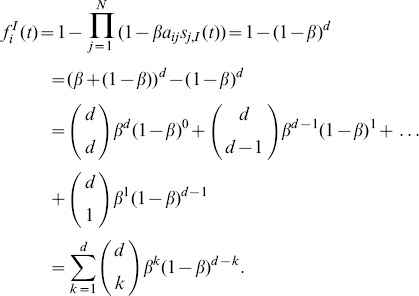
(2)


Equation (2) clearly shows that the probability 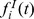 of receiving the infection from the neighbors is the sum of the probability that exactly 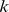 neighbors have successfully transmitted the infection to node 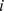, where 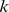 goes from 1 of the infected neighbors to all 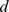 of the infected neighbors. The binomial coefficient takes into account all combinations of 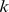 successful transmissions out of possible 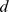 transmissions. The term 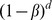 which corresponds to the event that no infected neighbor successfully transmitted the infection is canceled out in (2).

Here we stress that when exactly 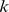 neighbors have successfully transmitted the infection, node 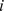 chooses only one of these, which means that the probability of receiving the infection from one of those 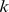 successful transmissions is 
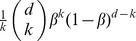
. Since there are 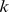 successful transmissions, the probability of receiving the infection from any one of those is 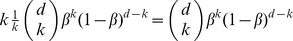, which is how the terms in the sum in (2) are obtained. This happens since all transmission events have the same probability 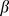 of occurring, which, in turn, leads to the possibility to write 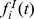 in the product form as in (1).

On the other hand, when more contagions spread in the network simultaneously and have different transmission probabilities, the fact that a node chooses only one of the 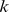 successfully transmitted, generally different, contagions makes it impossible to write the corresponding probability of infection 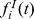 in the product form as in (1). The expression for 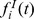, as we shall see in the following, will be analogous to (2), where all combinations of neighbors infected with different contagions will be included.

### Model description

We study the following discrete-time stochastic process for the spreading of multiple contagions in a network. The network is represented by a simple, undirected and connected graph 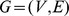 with node set 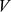 and link set 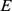. The graph's adjacency matrix is 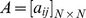, where 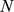 is the number of nodes in the network. We assume that every node 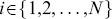 in the network is in a certain state 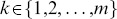. A state 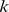 can represent the healthy state or the recovered state, as in the classical epidemic models, or it can denote that the node is infected with a certain contagion 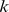. Node 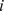
's state vector at time 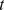 is represented by
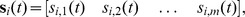
where 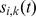, 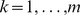, is a Bernoulli random variable indicating whether node 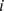 is in state 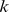. A node can be in only one of the model states at a time, meaning that only one component of the state vector is 1 and all others are 0 for each 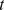. This is known as the 1-of-m coding scheme. The probability mass function corresponding to the state vector 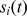 is



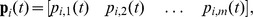
where 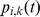 is the probability that node 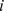 is in state 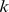 at time step 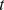. Naturally, 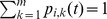 holds.

**Figure 2:**
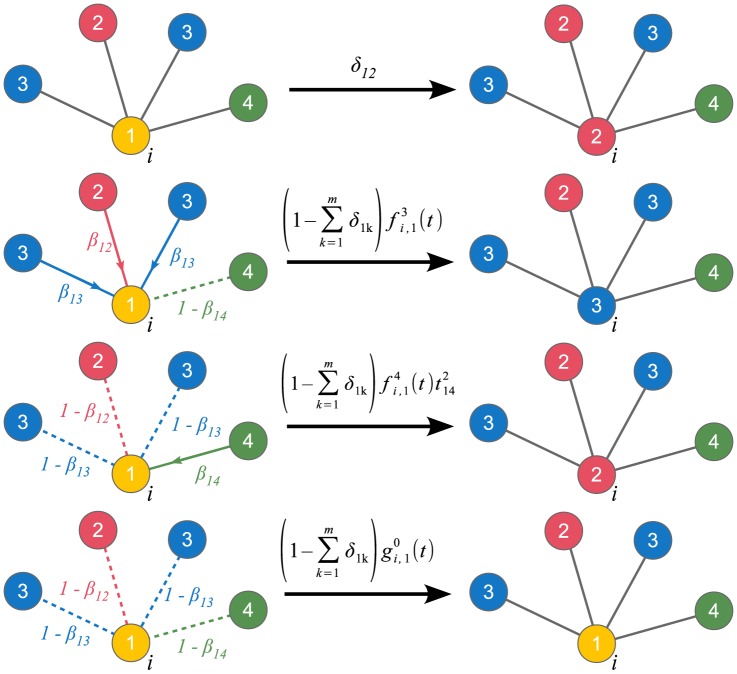
An illustration of the two mechanisms of state change of a node. The number of different states that exist in the network is 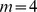. Solid colored arrows indicate successful state transmissions, i.e. infectious links, and dashed lines indicate an unsuccessful state transmission. The probabilities of the realized transmission events are depicted next to each line. Solid gray lines indicate that the nodes have not been in contact at the given time step; a spontaneous transition has taken place instead. From top to bottom panel, descriptions go as follows. Panel 1: node 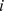 changes its state spontaneously to state 2 after previously having been in state 1. The probability of state change with this mechanism is 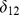. Panel 2: Node 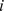 does not make a spontaneous transition, and changes its state as a result of getting infected with state 3 from its neighbors. Note that a neighbor in state 2 also makes successful transmission, however, node 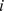 chooses state 3 transmitted from one of the other two successful transmissions. The probability of state change with this mechanism is 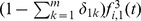, where 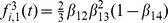. Panel 3: node 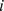 changes its state as a result of getting infected with state 4 from its neighbors, a contact which stimulates it to adopt state 2. The probability of state change with this mechanism is 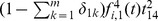, where 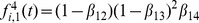 and 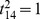. Panel 4: node 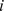 maintains its state since none of the two mechanisms of state change caused it to make a transition. The probability of this event is the product of the probability that no spontaneous transition occurs and no state is transmitted upon contact with the neighbors 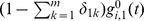, where 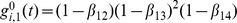.

The current state of a node can be changed by one of two mechanisms, as is illustrated in Fig. 2. The first mechanism is spontaneous transition to another state, without any contact with the neighbors. A node spontaneously abandons state 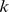 and transits to state 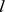 with probability 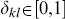. Note that 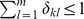 and that in general 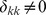. The process is equivalent to the rolling of a loaded 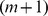-sided dice, where the outcome of the roll is the state to which node 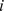 spontaneously transitions, and the 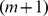
th side is the event that node 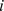 does not spontaneously change its state. This mechanism is analogous to node curing in epidemic models where nodes change from an infected to a healthy state. Should the node not make a spontaneous change, it proceeds with the second mechanism. The second mechanism is transition to other state due to contact with the neighbors and we also refer to it as the contact-induced state change mechanism. This mechanism is analogous to node infecting with a contagion from its neighbors. The transmission probability 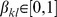, is the probability that a node in state 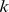 will change its state when contacting a neighbor in state 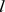. After a successful transmission, the receiving node may transit to state 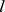 or, stimulated by the communication, adopt another state. To simplify the modeling process, we make the usual assumption that transmission events between separate pairs of nodes are independent of each other.

The model equations are
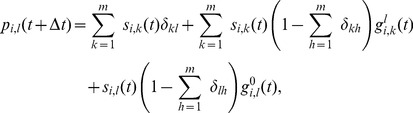
(3)where



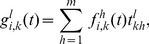
(4)

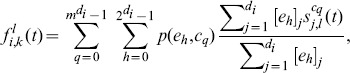
(5)




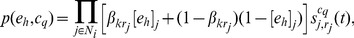
(6)

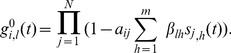
(7)


Equation (3) describes the time evolution of the probability that node 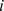 will be in state 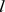 in the next time step. The first term of the right-hand side of (3) gives the probability with which node 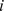 spontaneously transits to state 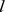 from its current state (first panel of Fig. 2). The second term encompasses the probability that, provided no spontaneous transition occurs, node 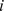 will adopt state 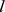 due to the second mechanism of state change (second and third panel of Fig. 2). The probability of this event is 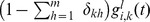, where 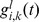 is the probability that node 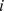 which is currently in state 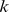 transits to state 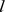 due to contact with the neighbors. The third term states the probability with which a node currently in state 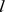 is not affected by any of the two mechanisms of state change. This event happens when the node does not spontaneously transit to any other state, and does not receive any other state or contagion when contacting the neighbors, the probability of which is 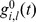 (fourth panel of Fig. 2).

The probability 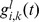 of state transmission, or infection with contagion 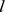, due to communication with the neighbors, given with (4), takes into account the two possible constructions of the contact-induced state change mechanism described previously in the introduction. The first one is typical for well-known epidemic models where node 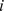 can transit to state 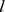 directly by contacting a neighbor in state 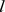, and this is depicted in the second panel of Fig. 2. The second one is encountered in social spreading processes, where the exposure of node 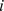 to another state 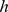 upon contact with the neighbors stimulates it to adopt state 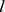. This is illustrated in the third panel of Fig. 2. Such is the case, for example, in the Maki-Thompson model where, upon contact of two informed nodes, one becomes a stifler since it loses interest in the rumor it possesses. Another, more complex, example would be the case where a single state is represented as a set of multiple distinct features, like in the language evolution models in [31], [32]. Upon successful infection from neighbor 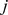, node 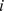 may copy only a fraction of the features from node 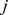 that it does not possess itself, effectively adopting a state that differs from node 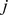
's state. A similar example is presented in [19], where exposure of an infected node to the other infection in the network causes it to transit to a third state which signifies that it possesses both infections simultaneously.

In (4), 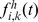 is the probability that state 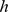 will cause change to node 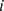 in state 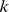 from any combination of its infectious neighboring links and is the analogue of (1) for the SIS model. 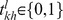 is an indicator variable which states whether the exposure of a node in state 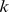 to state 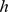 will stimulate it to adopt state 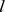. Note that when node 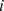 in state 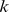 transits to state 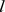 only by receiving it directly from its neighbors, 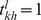 for 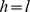 and 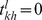 for all 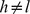. Hence, we have 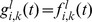, which is the most common case in the existing models. The sum in (4) goes over all states 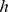 which can stimulate a node in state 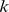 to adopt state 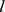. In essence, 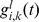 is a generalization of 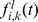 for the purpose of encompassing the second construction. A slightly more general case is the one when 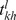 are parameters such that 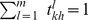 and 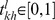. This makes 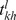, for 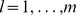, to act as weights to the transitioning states instead of indicator variables. Again, a good example would be the case with states which describe the possession of multiple features, where upon successful infection a node may have multiple possible courses of action, regarding the choice of copying individual features. We do not treat such cases in this paper.

The specific spreading process determines the form of 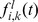. In this paper we concentrate on generalizing the reactive process as indicated in [12] for an arbitrary number of node states. As mentioned previously, this is the type of process most often to be found in the literature. In the reactive process, a node contacts all of its neighbors in every time step and tries to spread its current contagion to all of them, i.e. it tries to convince the neighbors to adopt the state that the node is currently in. As stated above, the probability of transmission 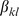 depends on the states 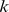 and 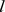 of the contacting nodes (receiving and sending node, respectively). The link over which a successful transmission has occurred is said to be *infectious* at the given time step. As transmissions are independent of each other, multiple infectious links may occur in a single time step at a receiving node. Most of the papers in the literature avoid this problem by assuming infinitesimally small time steps, virtually avoiding multiple simultaneous infection events. For node 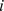 with a set of neighbors 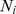, where 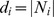 is the node degree, there are 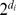 possible events as a result of whether each of the 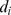 links becomes infectious or not. Hence, it is convenient to represent each such event 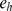, 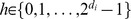, as a vector of length 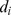 where each component 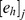 is equal to 1 if the link with the corresponding neighbor is infectious, and 0 otherwise. For example, the specific event which has happened to node 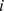 on Fig. 2, second panel, is represented by 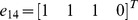. The numbering of the vectors 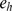 is given by the numerical (decimal) value corresponding to the binary number comprised of the components of 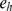, where the least significant digit in the binary number is the last component of 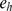. The probability of occurrence of event 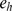, 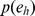, depends on the state of each neighbor and the state of the node 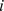. For node 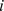 with degree 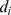 and a total of 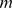 different states in the network, there are 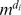 possible configurations (variations with repetition) of states that the neighbors of node 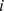 can be in. We denote each configuration with 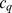, where 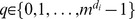. For example, the configuration of states on the second panel of Fig. 2 is 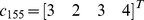. Analogously, the numbering of each configuration 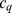 is given by the decimal number corresponding to the base-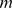 number comprised of the components of 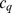 subtracted by one (for zero-based numbering purposes).

Now, the probability 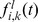 that node 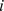 which is in state 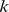 adopts state 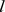 from any combination of its infected neighbors for the reactive process is given with (5). Equation (5) goes over every possible configuration of states 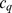 at the neighbors of node 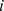 and every possible event 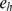. The terms 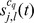 and 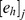 are used to filter only the combinations where state 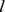 is involved. 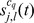 is simply the Bernoulli random variable which indicates whether node 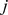 is in state 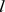 in the configuration 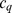. When there are multiple successful infectious links in 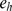, i.e. a total of 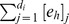 infectious links, node 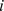 chooses one of the transmitted states to adopt, with uniform probability 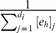.

In order to provide the expression (6) for 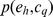 we denote the current state of each neighbor 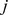 with 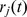. Since we make the assumption that a transmission between contacting nodes is independent of other transmissions in the network in the current or past time steps, the probability that event 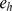 occurs at node 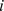 is simply a product of the probabilities that the respective links in the event have become infectious or not. 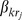 is the probability that the link with neighbor 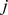 becomes infectious. The argument from 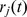 is omitted for brevity (we make the same omissions in the rest of the text).

Lastly, 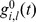 in (3), given with (7), is the probability that node 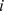 in state 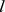 does not adopt any state from its neighbors, i.e. no infectious links occurred in the current time step.

The computational complexity of (5) is of order 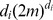. Due to the exponential dependence on the degree 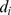 of node 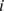, the probability 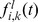 becomes numerically intractable for nodes of high degree. Therefore, further in the text we give an approximation for (5) which allows the application of the model for networks with high-degree nodes. We also speculate that the difficulty in finding a suitable expression for the probability 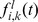 which is numerically feasible is the reason why a general model of this kind has not appeared so far.

The model has three types of parameters: the transmission probabilities 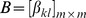, the probabilities of spontaneous transition 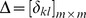, and the indicator variables 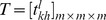. Although the number of parameters is large, for most of the models in the literature that we generalize, these matrices adopt a sparse structure. In the rest of the paper we continue with the implicit assumption we have made so far, that the parameters are the same for every node. However, node dependence of the parameter values is easily incorporated by making the parameters different for each node.

### Approximation

The complete derivation of the approximation, which is given in Section S1 of Text S1, leads to the following approximate expression for the probability 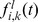 given with Eq. (5):
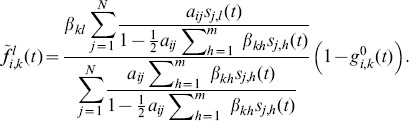
(8)


The derivation process was conducted while bearing in mind the compatibility with the deterministic counterpart, where instead of state vectors there are probability vectors. Hence, Eq. (8) can naturally be used for the deterministic case. As a result, it takes slightly complex form. However, we can make a simplification for the stochastic case. Let 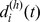 be the number of neighbors of node 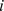 that are in state 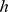; let 
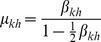
. Then Eq. (8) can be rewritten in a more compact form as
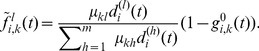
(9)


Observe that when 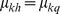, i.e. 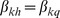, for all 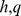, we have 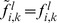 for all 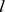. We stress that the term 
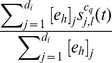
 in (5), which represents the fraction of neighbors in state 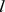, is primarily the term that is approximated. As already mentioned, for models where only one state is being transmitted by the contact-induced mechanism, this fraction is equal to 1 and the probability of receiving the state can also be written in the product form (1). The nonlinearities in the model arise from the product term in (6). In existing models for the spread of a single contagion, this product is most often linearized using a general form of the Weierstrass product inequality for the purpose of model analysis. Particularly, Eq. (1) is usually substituted by 
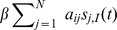
, an approximation which holds only for 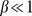.

An assessment of the accuracy of the approximation is presented in the section where we discuss our generalization of the 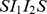 model and further in the section where we present an example numerical model. We compare the non-approximated and approximated version of the deterministic counterpart of our model, described in the following section. Additional assessments are also given in the supporting material (Section S2 of Text S1). Results indicate that the absolute value of the error is of order 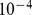 to 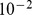 for a single node, depending on the specific scenario. Increasing the number of neighbors with one of the states produces a lower precision of the approximation when the state transmission probabilities are high (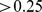) than when they are low. Also, the approximation usually overestimates the actual probabilities for states with high transmission probabilities when they interplay with states with low transmission probabilities, for which the actual probabilities are underestimated as a result. On the other hand, the approximation error is very low when the set of state transmission probability values has a small variance. An important result is that the error does not seem to accumulate over time and individual errors do not influence each other significantly. This has been observed in the 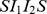 model and its altered version, as the predictions of the fixed points produced by the approximated version are in agreement with those produced by the non-approximated version. Further, the numerical example showed that the approximation is accurate throughout the time evolution even when the spreading dynamics showed oscillatory behavior. This was also the case for a network with high degree nodes.

Besides making the model numerically tractable, the approximation also allows for the estimation of the model parameters and their joint posterior distribution when data about the process being modeled are available. The joint posterior distribution of the parameters is a high-dimensional distribution which can be approximated by Markov Chain Monte Carlo sampling methods, such as the the Metropolis-Hastings algorithm. The posterior distribution is proportional to the likelihood of the observed data (in this case, the states of the nodes in the network at certain time steps), and the probability mass vectors for each node (obtained by running Eq. (10)) are required to calculate the likelihood. The probability mass vectors, in turn, depend on the probabilities 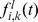 which can easily be calculated using the approximation 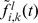. Using Bayesian inference, an analysis of any function of the parameters can be performed.

### The deterministic counterpart of the model and its continuous-time form

By applying the expectation operator to Eq. (3), and taking into account the assumption of independence of transmission events in the current or past time steps, as well as the fact that 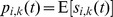, from Eqs. (3)–(7) a deterministic discrete-time version of the model is obtained, which is basically a discrete-time nonlinear dynamical system:
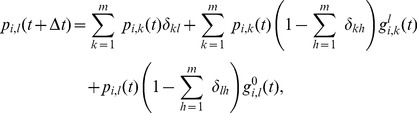
(10)where



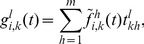
(11)

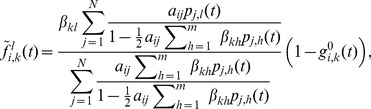
(12)




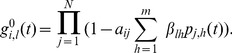
(13)


For brevity, we only presented the approximated equations of the model here, while the non-approximated version is analogous to Eq. (5). This deterministic discrete-time version of the model describes the dynamics of expected-value quantities of the stochastic model. Instead of running just one stochastic realization with the stochastic version of the model, or running sufficiently many to produce average results, one can use the deterministic model to obtain the average dynamics of the spreading process and make predictions as to its future state. Hence, we expect that the deterministic results will be comparable with those obtained from the mean values of the stochastic realizations in the limit of large network sizes. Furthermore, it also allows for a simpler analysis of the dynamical behavior of the model, which we leave for future work. For example, using a classical result for the weak ergodicity of time-inhomogeneous Markov chains by Wolfowitz [33], one can determine conditions by which the deterministic version of the model has a globally stable fixed point, which means that the average dynamics of the stochastic model stabilize. Such an analysis is given in [20], for instance.

In order to obtain a deterministic continuous-time, i.e. differential equation, model, the product in (13) is typically linearized, which holds for 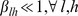. Rearranging the terms in Eq. (10) in a way that one can calculate the limit 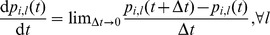, the deterministic differential equations for the evolution of the probability mass function of node 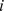 are obtained (derived in detail in Section S3 of Text S1):
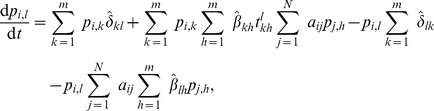
(14)or in the case when 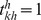, 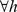, it reads
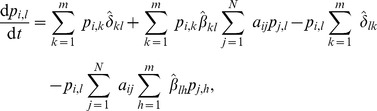
(15)where each parameter that represents a probability in the discrete-time model is substituted by its respective rate (e.g. 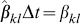, for time step 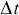). The first and the second term on the right hand side of Eqs. (14) and (15), which increase the rate of change of 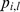, correspond to transitions from other states to state 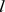 due to the spontaneous and contact-induced state change mechanism respectively, while the other two terms correspond to the respective state transitions in the opposite direction. One can also note that the second term on the right hand side of Eq. (15) has a simpler form than the corresponding term from the discrete-time case (Eqs. (10)–(13)). Specifically, the continuous-time one depends only on the components of the neighbors' probability vectors that correspond to state 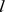. This is due to the infinitesimally small time step size which prevents the occurrence of more than one transmission event in any given time instance. Equation (15) written in matrix form reads
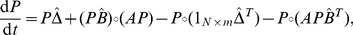
(16)where the operator 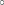 represents the Hadamard (or element-wise) product. The matrix 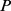 has the probability vectors of the nodes as rows, while the matrices 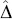 and 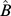 have the rates of the spontaneous and the contact-induced transition mechanism as elements, respectively. 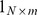 is an all-ones matrix with 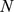 rows and 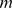 columns. 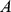 is the adjacency matrix of the network, as mentioned earlier.

## Special Cases of the Model

**Figure 3:**
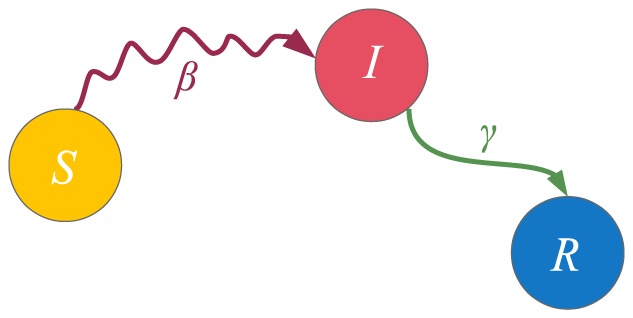
State diagram for the SIR model. The diagram shows the dynamics of a single node. A susceptible node can become infected by contacting its infected neighbors, with transmission probability 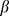. On the other hand, infected nodes spontaneously recover with probability 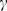 and they remain permanently immune to the infection. The contact-induced transition mechanism is represented by a curvy arrow, whereas the spontaneous transition mechanism is represented by a less curvy arrow.

**Figure 4:**
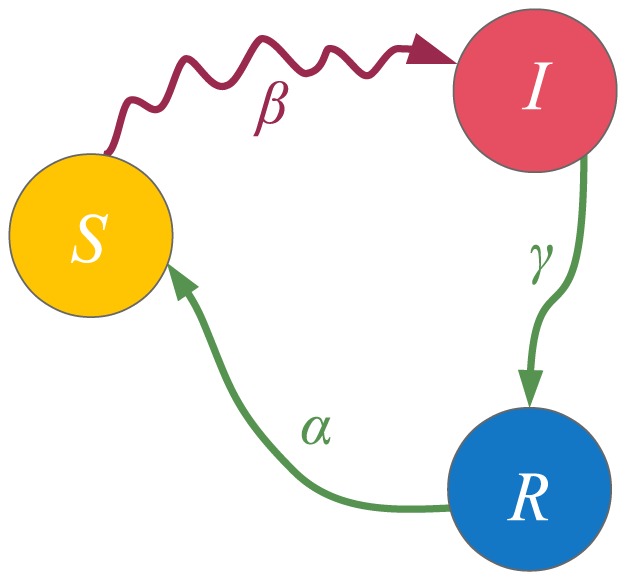
State diagram for the SIRS model. The diagram shows the dynamics of a single node. A susceptible node can become infected by contacting its infected neighbors, with transmission probability 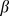. On the other hand, infected nodes spontaneously recover with probability 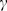. However, they only obtain temporal immunity which they lose with probability 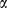. The contact-induced transition mechanism is represented by a curvy arrow, whereas the spontaneous transition mechanism is represented by a less curvy arrow.

**Figure 5:**
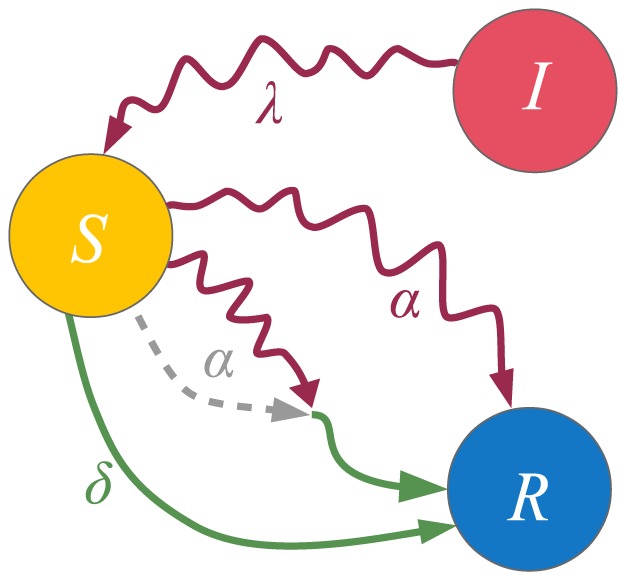
State diagram for the Maki-Thompson model of rumor spreading. The diagram shows the dynamics of a single node. An ignorant node can become a spreader by contacting its neighbors that spread the rumor, with rate 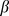. On the other hand, spreader nodes become stiflers by contacting other spreaders or stiflers with rate 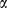, or by spontaneously transitioning to the stifler state with rate 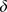. The contact-induced transition mechanism is represented by a curvy arrow, whereas the spontaneous transition mechanism is represented by a less curvy arrow. The dashed arrow denotes that 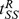, i.e. spreader becomes stifler by contacting other spreaders with rate 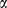.

**Figure 6:**
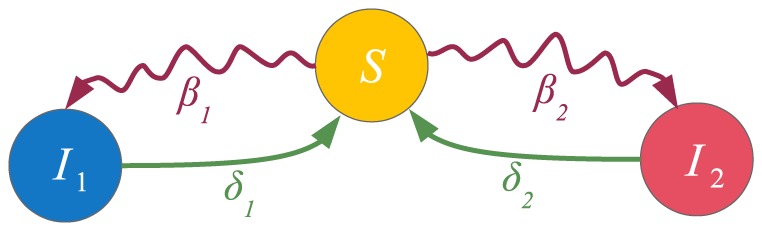
State diagram of a node for the 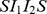 model. The diagram shows the dynamics of a single node. Curvy arrows depict state change due to contact with the neighbors, while less curvy arrows depict spontaneous state change.

In this section we present some well-known models which the proposed model generalizes. Since the model equations (3) constitute an inhomogeneous Markov chain for each node, we give the state diagrams of the Markov chains for each of the models in Figs. 1–6. The models discussed are the widely known SIS, SIR and SIRS epidemic spreading models; the Maki-Thompson rumor spreading model; and the 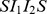 model where two contagions spread concurrently in the network, for which we also assess the accuracy of our approximation. We present the natural extension of our model on multiplex networks, as well. Although the model equations appear to be complex in the general case, we can observe that they reduce to much simpler ones due to the sparsity of the parameter matrices of the presented models. For the discrete-time forms of each of the models, the parameters which represent probabilities are used in Eq. (3) and Eq. (10), while for the continuous-time forms the respective rate parameters are used in Eq. (14). Other models which we have met in the literature and which the proposed model generalizes are given in [19], [20].

### The epidemic spreading models

#### The SIS model

The SIS model is described in the previous section. Its state diagram is presented in Fig. 1. Recall that the state of node 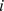 at time 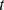 is described by a state vector of length 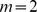 for the SIS model, specifying whether it is in the susceptible (S) or infected state (I). The model equations (3) for the evolution of the probability mass function become:
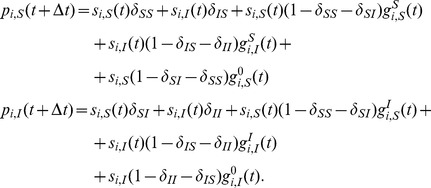
(17)


Now, since the contact-induced transition mechanism of the SIS model is infection spread from infected nodes to susceptible nodes, and the spontaneous transition mechanism involves spontaneous transition only from the I to the S state, the parameters of the model are:
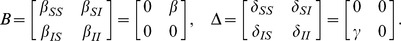



The parameters for the continuous-time case, 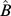 and 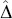, are defined analogously. Note that in the SIS model no interaction with a given state stimulates a node to adopt a state other than the given one involved in the interaction. Therefore, 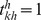, 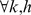, so we have 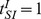. This means that 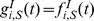. Also, as noted previously, when a node can be affected by only one infection through the contact-induced mechanism, no approximation is needed to write out (5) and 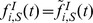 can be written in the product form as in (2). This gives 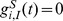, 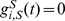, 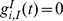, and 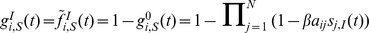. Taking all of the aforementioned into account, we find that (17) reduces to the known equations for the SIS model:
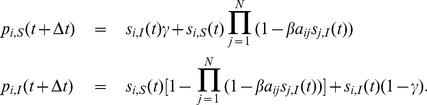
(18)


Similarly, using the parameters 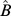 and 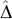 with Eq. (14), we obtain the continuous-time equations of the SIS model:
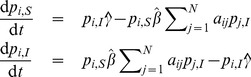
(19)


#### The SIR model

The SIR, or susceptible-infected-recovered model, is used to describe the spread of an infection for which permanent immunity is obtained after the end of the infectious period. The state diagram that describes its Markov chain is given in Fig. 3. A node can be in one of three states: the susceptible (S), infected (I) and recovered (R) state, hence 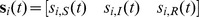 for a given node 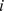. The general model equations (3) for the SIR model are:
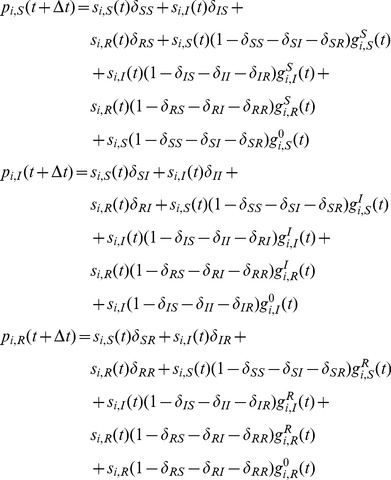
(20)


According to the description of the model, the parameters of the model are
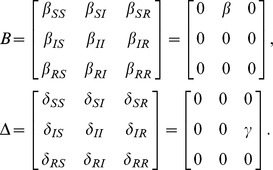



The parameters for the continuous-time case, 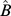 and 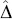, are defined analogously. As in the SIS model, the contact-induced transition mechanism in the SIR model exists only in the interaction between susceptible and infected nodes, and the number of infected nodes is increased as a result. This implies 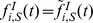. No interaction with a given state stimulates a node to adopt a state other than the given one involved in the interaction. This means that 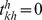 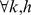, so we have 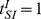. Similarly to the SIS model, we have that 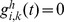, 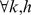, except for 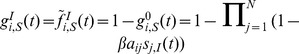. Taking all of the aforementioned into account, the equations (20) reduce to
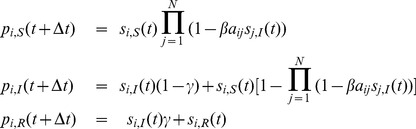
(21)


Similarly, using the parameters 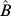 and 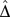 with Eq. (14), we obtain the continuous-time equations of the SIR model:
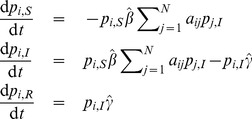
(22)


#### The SIRS model

The SIRS, or susceptible-infected-recovered-susceptible model, is used to describe the spread of an infection for which temporary immunity is obtained after the end of the infectious period. The state diagram that describes its Markov chain is given in Fig. 4. It is very similar to the SIR model, the only difference being the addition of a spontaneous transition link from the recovered to the susceptible state that describes the temporary immunity. Having the same states as the SIR model, the general model equations (3) for the SIRS model coincide with those of the SIR model, Eq. (20). As mentioned earlier, the only difference in the model parameters is that 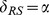 in the SIRS model, whereas 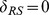 in the SIR model. Hence, the equations (3) reduce to
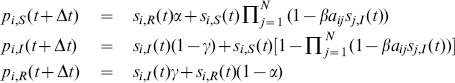
(23)


Similarly, the continuous time equations of the SIRS model read
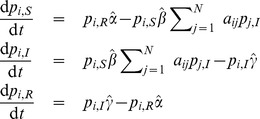
(24)


### The Maki-Thompson model for rumor spreading

The Maki-Thompson model [34] is a popular variant of the classical model of rumor spreading of Daley and Kendall [35]. In both models, which operate in continuous time, each node can be in one of three different states: ignorant (I), spreader (S) and stifler (R). Nodes in state I are uninformed, or ignorant, of the given rumor and are thus susceptible to it. Nodes in state S actively spread the rumor, while nodes in state R are aware of the rumor, but they have lost interest in it and no longer spread it. When an ignorant node contacts a spreader, it becomes a spreader as well with rate 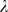. If, on the other hand, a spreader contacts a stifler or another spreader, it becomes a stifler at a rate 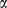. The two models, Maki-Thompson and Daley-Kendall, differ in the contact mechanism. The Daley-Kendall model adopts a pair-wise contact mechanism, i.e. for a given neighboring pair only one communication is assumed, which can affect both nodes simultaneously. For example, two neighboring spreaders either both become stiflers due to a single successful transmission or otherwise they both remain spreaders. Our model does not generalize such models. On the other hand, the Maki-Thompson model adopts a directed contact mechanism, i.e. only the node which initiates the contact can change its state. Hence, separate contacts will be initiated for both directions in every time step for the discrete time case, as we work with a reactive process. Both models assume a homogeneously mixed population or an undirected network; however, the Maki-Thompson model can easily be applied on directed networks as well. Also note the resemblance between the ignorant, spreader and stifler state in the Maki-Thompson model and the susceptible, infected and recovered state in the SIR model. The difference with the epidemic models is the part of the contact-induced transition mechanism which comes from contacts initiated by the spreaders. The mechanism of spontaneous transition to another state is absent from the original versions of both models.

A more recent study has supplemented both models with a mechanism of spontaneous rumor forgetting with rate 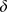 by which spreader nodes can also become stiflers [36]. The state diagram describing the Markov chain of this version of the Maki-Thompson model is given in Fig. 5. Here we show that the proposed model reduces to the Maki-Thompson model with rumor forgetting. Moreover, we recover the differential equations that have actually been used in [36]. The state of a node is described by a vector of length 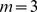: 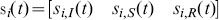, and so is the probability mass function which gives the probability of being in each state. Regarding the model parameters, note that 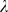, 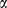 and 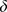 are rates, and the corresponding probabilities are obtained by multiplying these with the length of the time step 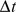 in which a contact occurs. Hence, the parameters are
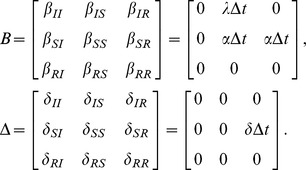



This means that 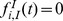, 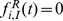, 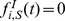, 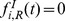, 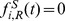 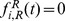, while 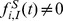, 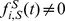 and 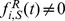 in general. The contact-induced transition mechanism in the Maki-Thompson (and Daley-Kendall) model describes the increase in the number of both the S–nodes and the R–nodes. The increase in the number of spreaders is achieved by the interaction of nodes in states I and S, which is an infectious spread of the S state. The increase in the number of stiflers occurs because of interactions between nodes in states S and R, which can be viewed as an infectious spread of the R state, and because of interactions between nodes in state S, where the contact stimulates a node to adopt the R state. Therefore, 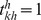, 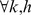, except for 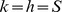, where 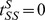 and 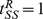, which results in 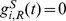, 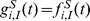 and 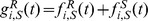. Taking all of the aforementioned into account, the equations for the Maki-Thompson model become
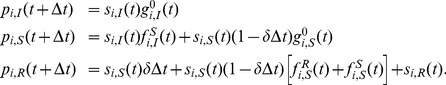
(25)


Now note that although the contact-induced mechanism practically describes two states spreading in a network, the two of them cannot affect a node simultaneously, i.e. do not have to compete for a node, since I–nodes can only transit to S–nodes via the contact-induced mechanism, and S–nodes can only transit to R–nodes. In effect, from a node's viewpoint, each state spreads as if it is the only contagion in the network. Thus, there is no need to approximate (5) and 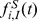, 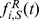 and 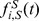 can be written in the product or sum form as in (2):
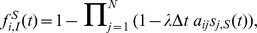


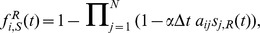


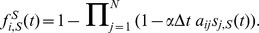



The probabilities 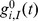 and 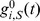 of not changing the current state given with (7) become
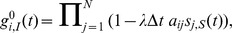


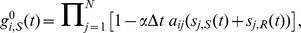
and 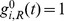, which gives rise to the term 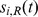 in the last equation of (25).

Regarding the continuous-time case, using the rate parameter matrices, 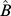 and 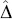, with the matrix 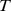 and Eq. (14), we obtain the following deterministic differential equations for the evolution of the probability mass function of node 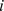, which are the same as the ones from [36]:
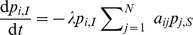


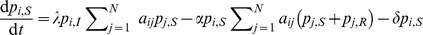


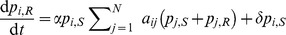



Further, if we use a homogeneous mean-field approximation, which means that we assume that every node 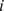 has the same degree 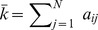, and hence the same dynamical behavior, i.e. 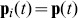 for all 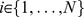, one obtains
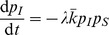


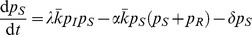


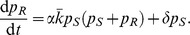



Lastly, the original version of the Maki-Thompson model is obtained for 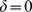.

### The 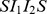 model

As a suitable example for assessing the accuracy of our approximation, we present our discrete-time generalization of the continuous-time 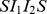 model [26]. The state diagram that describes a node's transition probabilities is presented in Fig. 6. In this model, there is a competition between two different contagions (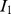 and 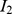, or states 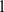 and 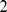, respectively), in the sense of which contagion will infect a given susceptible node (in state 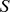, or state 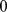). Their respective transmission probabilities are 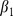 and 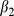. Also, once infected, a node can become susceptible again with a probability 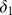 or 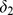, with respect to 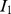 and 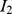. Taking all of this into account, the discrete-time model equations (10) for the deterministic 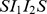 are
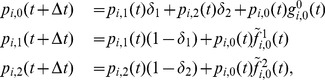
(26)where
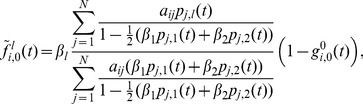
(27)for 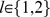, and



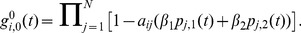
(28)We can derive the corresponding continuous-time model equations (15) as well, which read
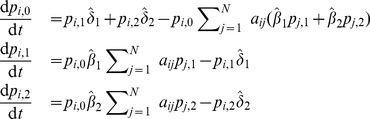
(29)


Equation (29) coincides with the one derived in [26].

**Figure 7:**
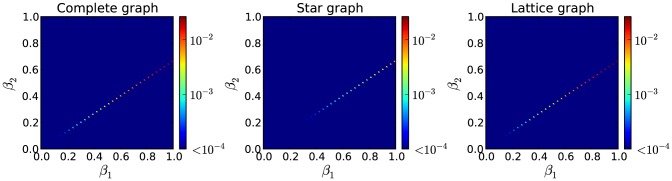
Comparison of the macroscopic fixed point values produced by the approximated and non-approximated version of the discrete-time generalization of the 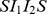 model. The norm of the error vector, whose components are the differences between the macroscopic fixed point values (percentage of nodes in each state) of both versions, is calculated for each combination of the parameters 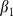 and 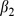. 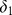 and 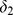 parameters are fixed at 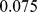 and 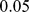, respectively. Three different graphs are examined with three random initial state assignments of the nodes: a complete and a star graph with 6 nodes, and a lattice graph with periodic boundary conditions of 9 nodes. Our approximated version produces the same fixed points as the non-approximated version, except for the line 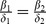 that depicts the area where there is no clear winner.

In order to assess the accuracy of the approximation of our model for the discrete-time case (Eqs. (10)–(13)), we compare it with the actual non-approximated version on several small networks (complete, star, ring and lattice graphs). The limitation on the network size comes as a result of the computational complexity of the non-approximated version. We calculate the macroscopic fixed points, i.e. percentage of nodes in each of the states, produced by both versions of the model for a given set of parameters. The norm of the error vector for every combination of 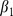 and 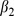 is calculated. As the approximation refers to the contact-induced state change mechanism, we keep 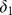 and 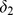 fixed at 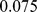 and 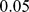, respectively. The results show that our approximation produces the same macroscopic fixed points as the non-approximated version, for almost every combination of 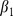 and 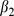. This can be expected, since in this model, as shown in [26], there is always a clear winner except for the case when 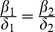, which is the line where our approximation does not produce zero error. This is shown in Fig. 7, for the complete graph, the star graph and the lattice graph.

**Figure 8:**
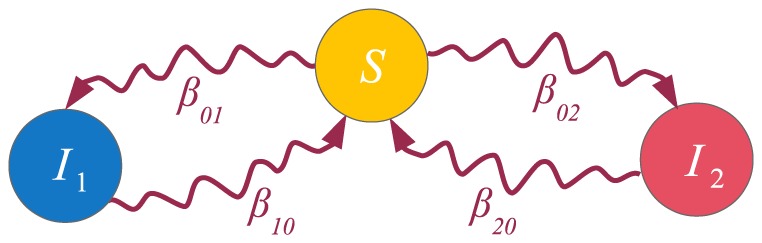
State diagram of a node for the altered 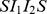 model that operates only with the contact-based mechanism. The diagram shows the dynamics of a single node. Curvy arrows depict state change due to contact with the neighbors.

**Figure 9:**
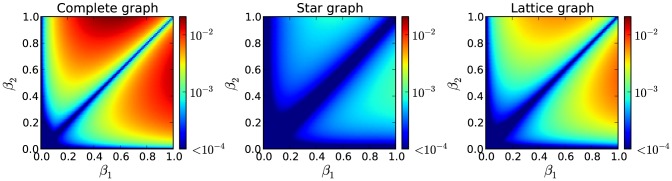
Comparison of the macroscopic fixed point values produced by the approximated and non-approximated version of the discrete-time generalization of the altered 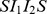 model that operates only with the contact-based mechanism. The norm of the error vector, whose components are the differences between the macroscopic fixed point values (percentage of nodes in each state) of both versions, is calculated for each combination of the parameters 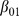 and 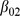. 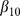 and 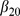 parameters are fixed at 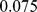 and 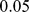, respectively. Three different graphs are examined with three random initial state assignments of the nodes: a complete and a star graph with 6 nodes, and a lattice graph with periodic boundary conditions of 9 nodes. Results show that the approximation is accurate in the regions where both 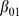 and 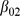 are small and also where they are roughly the same.

A model with more complex behavior can be easily constructed, and it will help in assessing the accuracy of our approximation. We alter the aforementioned 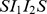 model by replacing the spontaneous transition links with contact-based transition links. The state diagram of this model is presented in Fig. 8. The process that we model can be thought of as a competition between two political parties (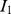 and 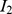). As before, a person that does not support either party can become a supporter by contacting other supporters of one of the parties. Also, a voter can decide not to vote by contacting other such persons, having previously supported one of the parties (unlike the previous model, where such state change can only be made spontaneously). The macroscopic fixed points were compared for the approximated and the non-approximated version of our model, for every combination of 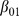 and 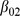. Similarly as before, we kept 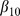 and 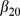 fixed at 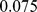 and 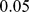, respectively. In this model a clear winner is not always found. The approximation refers to the contact-induced state change mechanism of the susceptible node, the same as in the original 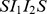 model. The results of the comparison between both versions, presented in Fig. 9, demonstrate how much the individual approximation errors influence each other and accumulate over time. They clearly indicate the regions where our approximation works best. For relatively comparable values as well as for small values (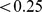) of both of the parameters 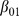 and 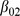 our approximation produces a negligibly small error on every graph. We find these results satisfactory as working within these regions has been standard practice in the literature. The error in the other regions is low as well. The results for the ring graph are not shown, since the error is significantly smaller than the errors for the other graphs.

### An example model for the spread of three innovations

**Figure 10:**
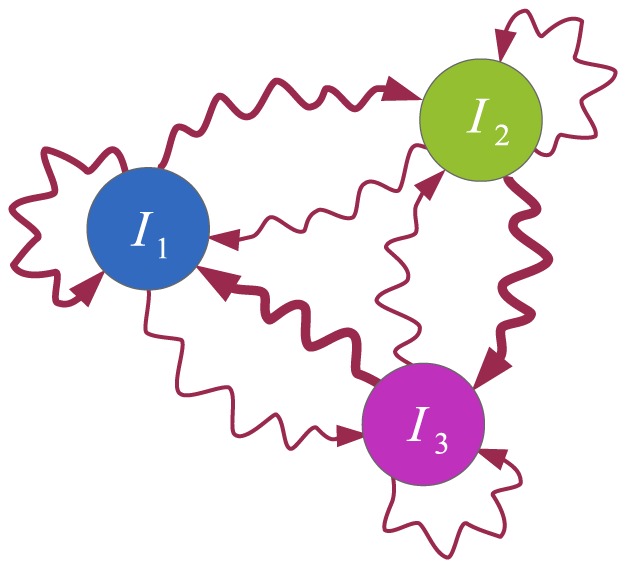
State diagram of a node for the 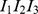 model that operates only with the contact-based mechanism. The diagram shows the dynamics of a single node. Curvy arrows depict state change due to contact with the neighbors. The arrow notations are omitted for brevity. The arrow widths are proportional to the parameter values of the contact-based transitions between the respective states.



While the previous example model was suitable for presenting the regions where our approximation very closely matched the non-approximated deterministic version of our model, the macroscopic behaviors always converged to a fixed point where one of the states had vanished from the system. We observed that the deterministic behaviors did not show oscillations, due to the simplicity of the model. In order to test the approximation on a system with oscillatory behavior, we introduce an example model for the spread of three innovations, whose state diagram is shown in Fig. 10. Here, each node possesses one of the three innovations (there is no susceptible state in this model), and contact-induced transitions can occur between any two states, including transitions to the same state. The parameter matrix 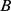 that was used is

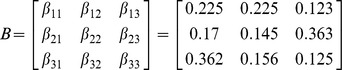



**Figure 11:**
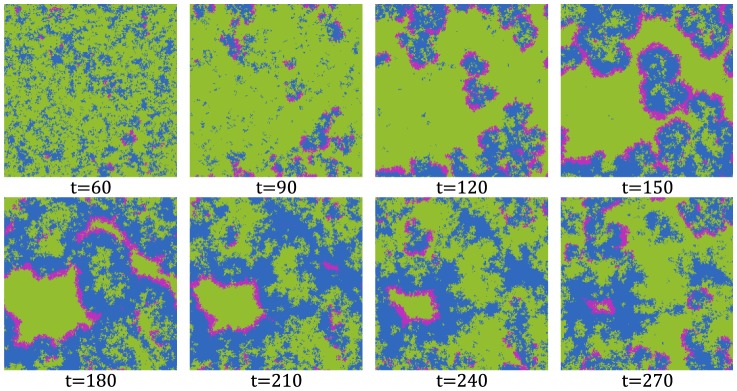
Snapshots of one sample execution of the 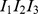 model on a lattice network with periodic boundary conditions of 65536 (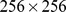) nodes. The snapshots were taken at different time steps, as indicated below each individual snapshot. Cyclic-like behavior is clearly seen.

Relatively large values were selected to establish whether our approximation works well even for the parameters where the error was not zero in the previous assessments (Fig. 9). Also, as mentioned earlier, they were selected to create oscillatory, i.e. cyclic-like, behavior. More specifically, should we leave only the maximal element of each row of the matrix 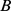, the respective state diagram will depict a cycle graph. The link widths in the state diagram (Fig. 10) are proportional to the corresponding parameters from the matrix 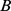 as well. A sample execution on a lattice network with periodic boundary conditions of 65536 (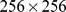) nodes is presented in Fig. 11, that demonstrates the oscillatory behavior. Snapshots of the system state at eight different time steps are given.

**Figure 12:**
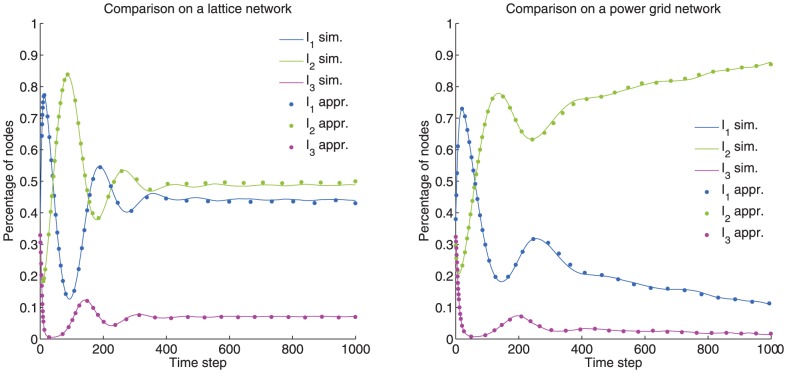
Comparison of the macroscopic behavior simulated by the approximated and non-approximated stochastic version of the model. Percentage of nodes in each of the states is displayed for each time step. The left panel shows the comparison results on a lattice network with periodic boundary conditions of 16384 (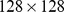) nodes. The right panel shows the comparison results on a power grid network of 4941 nodes and 13188 links, whose highest degree is 19. The results were produced by averaging over 1000 executions. The markers display the execution results of the approximated version, while the lines display the results of the non-approximated version, i.e. the actual model simulations. For brevity only 40 markers are displayed for each state.

Due to the large number of parameters in this model, a comparison of the fixed point values is inappropriate. However, another suitable way for assessing the accuracy of our approximation is to compare the actual simulations of the stochastic model, which are described by the non-approximated version of the model (Eqs. (3)–(7)), with the approximated version of the model (Eq. (9)). While comparing the non-approximated and approximated deterministic models is not possible for large networks and networks with high-degree nodes (due to the computational complexity of the non-approximated version of the model), it is easily done with the stochastic model. For a lattice network with periodic boundary conditions of 16384 (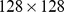) nodes, the comparison results are shown in Fig. 12, left panel. They were produced by averaging over 1000 executions. The markers display the execution results of the approximated version, while the lines display the results of the non-approximated version, i.e. the actual model simulations. Although we selected relatively large values for the parameters, our approximated version matched the non-approximated version very closely. To prove that this is also the case for networks that have nodes with large degrees, we performed the same comparison on a power grid network [37] of 4941 nodes and 13188 links, whose largest degree is 19. Results, shown in Fig. 12, right panel, again indicate that the approximation works well. At last, we performed similar comparisons on models with smaller parameter values, and the results, which we do not present here, showed that the approximated version matched the non-approximated version even better.

### Spreading in multiplex networks

**Figure 13:**
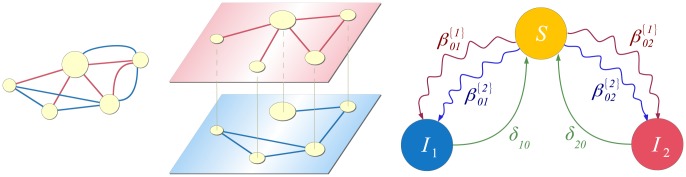
A multiplex network. Different link types correspond to different layers in the multiplex network. We assume that transmission probabilities depend on the link type, i.e. each contagion or state propagates differently over each layer. This is depicted by coloring the contact-induced transition mechanism links differently for each separate layer. In the adaptation of the 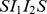 model for multiplex networks both contagions spread only on their respective layers. Hence, we have 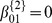 and 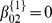.



To demonstrate the broad applicability of our model, we present the multiplex adaption of our model as a special case. A multiplex, or composite network, is a network whose nodes are interconnected with various types of links (Fig. 13, first panel) [38]. Each link type corresponds to a different layer in the network (Fig. 13, second panel), however, a node can only be in one single state over all layers in a given time step. Depending on how the multiplex network is defined, different variations may exist, e.g. a node's state can be depicted by a separate state for each layer. We focus on the single state case. As before, multiple contagions or states can spread in the network. However, the transmission probabilities between the states are layer-dependent, i.e. each of the contagions spreads differently on a different layer. Specifically, we have multiple adjacency matrices 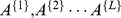 that describe the connectivity in each of the 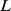 layers of the multiplex network. We also have multiple contact-induced transition mechanism matrices 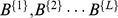, one for each layer, respectively, as shown in Fig. 13, third panel. Hence, the multiplex spreading equation for the probability that node 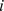 will be in state 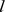 aligns with Eq. (10) with

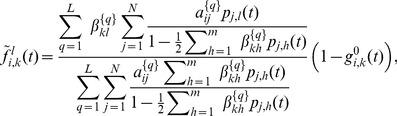
(30)and



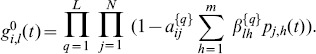
(31)Analogously, the differential equations read
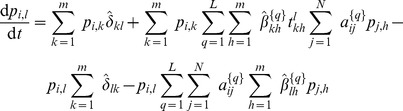
(32)


As can be seen, the model is powerful enough to capture spreading in multiplex networks. One example of such spreading is the multiplex variant of the 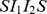 model [27]. There, two contagions (or memes) spread on two separate layers of the multiplex network. Mutual exclusivity is assumed between the contagions, i.e. a node infected with one of the contagions is immune to the other. The state diagram describing the model is presented in Fig. 13, third panel, and it coincides with the previous example. However, in this case, an infection has zero transmission probability on the layers it does not spread on. Hence, 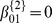, 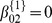, 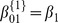 and 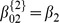; 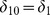 and 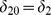. As a result, the equations (30) and (31) reduce to
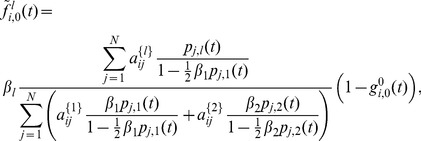
(33)for 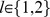, and



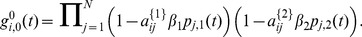
(34)The corresponding differential equations read
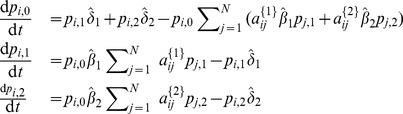
(35)


## Conclusions

In summary, this work has two main contributions. Firstly, we have proposed a general model for the spread of an arbitrary number of infections or contagions on networks, in which several contagions can simultaneously compete to infect a node. The model is stochastic and runs in discrete time, since difference equations are used to describe the evolution of the probability of being in each state. It can describe the spread of not only biological infections, but also social contagions such as information and rumors, cultural characteristics such as innovations and languages, and other systemic entities whose spread on networks can be described by the two mechanisms of state change incorporated in the model, which we refer to the spontaneous and the contact-induced state change mechanism. The first mechanism describes spontaneous transition to another state, and corresponds to the curing mechanism in classical epidemic models, while the second mechanism describes infection with other contagions or states due to contact with the neighbors, and corresponds to the spreading process in classical epidemic models. Secondly, an essential step for making the model applicable for simulations on networks is that we use the approximation (8) for the exact probability (5) that a node will adopt a specific state from its neighbors, which may possess any of the states in the network. The approximation showed high accuracy in most of the tested cases.

The proposed model generalizes classical epidemic models such as the SIS, SIR and SIRS models. Additionally, the contact-induced state change mechanism in the model accounts for spreading processes where the interaction with a given state can stimulate a node to adopt a state other than the one that is being interacted with. This extends the type of processes that can be modeled beyond epidemic spreading, and so the model also generalizes, for example, the Maki-Thompson rumor spreading model, a popular variant of the well-known Daley-Kendall rumor spreading model. The model allows for the transition to an equivalent discrete- or continuous-time, deterministic model of difference or differential equations, respectively, describing the spreading process which is being modeled. This may be useful for the study and comparison of stochastic and deterministic models of the same process. Spreading dynamics on multiplex networks is naturally captured by the model, as well.

## Supporting Information

Text S1 **Supporting text.** Section S1: Derivation of the approximation. Section S2: Assessment of the accuracy of the approximation. Section S3: Derivation of the continuous-time form of the deterministic counterpart of the model.
(PDF)
