# Supplementary material for: Modeling the Spread of Multiple Concurrent Contagions on Networks
Source: PLoS One. 2014 Jun 12;9(6):e95669. doi: 10.1371/journal.pone.0095669 (PMC4055576; doi:10.1371/journal.pone.0095669)
Supplement: Text S1 — Supporting text. Section S1: Derivation of the approximation. Section S2: Assessment of the accuracy of the approximation. Section S3: Derivation of the continuous-time form of the deterministic counterpart of the model. (PDF) [file pone.0095669.s001.pdf]

SUPPORTING TEXT S1

Modeling the Spread of Multiple Concurrent Contagions on Networks

Angel Stanoev[1], Daniel Trpevski[1], Ljupco Kocarev[1,*]
1 Macedonian Academy of Sciences and Arts, Skopje, Macedonia
∗ E-mail: lkocarev@manu.edu.mk

S1 Derivation of the approximation

Here we derive the approximation for Eq. (5) in the main text of the paper. At the beginning we make use of a simple example for the purpose of clarity. At first, consider the left panel of Figure S1, which shows node i connected to four neighbors, each of which is in one of four different states. For this node there are 2^4 possible events with respect to the state transmissions which can occur in a single time step. For the particular configuration $c_{27} = [1\ \ 2\ \ 3\ \ 4]^T$ of neighboring states, the event $e_9 = [1\ \ 0\ \ 0\ \ 1]^T$ depicted on the right panel of Fig. S1 has a probability of realization given with $p(e_9, c_{27}) = \beta_{k1}(1-\beta_{k2})(1-\beta_{k3})\beta_{k4}$. Assume that we are interested in finding $f^l_{i,k}(t)$ for $l \in \{1, 2, 3, 4\}$. Without loss of generality, we first compute the probability $f^1_{i,k}(t)$ that node i, which is in state k, will adopt state 1 from its neighbors at time step t. We depict with r_j the state of node j in a given configuration. So, $s^{c_{27}}_{j,r_j}(t) = 1$, $\forall j \in N_i$. Further, for each event e_h, Eq. (6) will be of the form

$$p(e_h, c_{27}) = \prod_{j \in N_i} [\beta_{kr_j}[e_h]_j + (1 - \beta_{kr_j})(1 - [e_h]_j)].$$

Since state 1 is present only at one of the neighbors, only the events e_8 - e_{15}, which account for a successful transmission of state 1, will be present in $f^1_{i,k}(t)$. Except in the case of event e_8, all other events have more than one successful state transmission from the neighbors, and from those, node i can adopt state 1 with uniform probability. Hence, $f^1_{i,k}(t)$ is

$$\begin{aligned}
f^1_{i,k}(t) &= p(e_8) + \frac{1}{2}p(e_9) + \frac{1}{2}p(e_{10}) + \frac{1}{2}p(e_{12}) \\
&+ \frac{1}{3}p(e_{11}) + \frac{1}{3}p(e_{13}) + \frac{1}{3}p(e_{14}) + \frac{1}{4}p(e_{15}) = \\
&= p([1\ 0\ 0\ 0]^T) + \frac{1}{2}p([1\ 0\ 0\ 1]^T) + \frac{1}{2}p([1\ 0\ 1\ 0]^T) + \frac{1}{2}p([1\ 1\ 0\ 0]^T) \\
&+ \frac{1}{3}p([1\ 0\ 1\ 1]^T) + \frac{1}{3}p([1\ 1\ 0\ 1]^T) + \frac{1}{3}p([1\ 1\ 1\ 0]^T) + \frac{1}{4}p([1\ 1\ 1\ 1]^T) = \\
&= \beta_{k1}\Big[(1-\beta_{k2})(1-\beta_{k3})(1-\beta_{k4}) + \frac{1}{2}(1-\beta_{k2})(1-\beta_{k3})\beta_{k4} + \\
&+ \frac{1}{2}(1-\beta_{k2})\beta_{k3}(1-\beta_{k4}) + \frac{1}{2}\beta_{k2}(1-\beta_{k3})(1-\beta_{k4}) + \\
&+ \frac{1}{3}(1-\beta_{k2})\beta_{k3}\beta_{k4} + \frac{1}{3}\beta_{k2}(1-\beta_{k3})\beta_{k4} + \frac{1}{3}\beta_{k2}\beta_{k3}(1-\beta_{k4}) + \frac{1}{4}\beta_{k2}\beta_{k3}\beta_{k4}\Big].
\end{aligned} \tag{S1}$$

After some rearrangements, it is readily obtained that

$$f^1_{i,k}(t) = \beta_{k1}\Big[1 - \frac{1}{2}\beta_{k2} - \frac{1}{2}\beta_{k3} - \frac{1}{2}\beta_{k4} + \frac{1}{3}\beta_{k2}\beta_{k3} + \frac{1}{3}\beta_{k2}\beta_{k4} + \frac{1}{3}\beta_{k3}\beta_{k4} - \frac{1}{4}\beta_{k2}\beta_{k3}\beta_{k4}\Big]. \tag{S2}$$

As noted in the main text of the paper, Eq. (S2) and, in general, Eq. (5) is of combinatorial nature

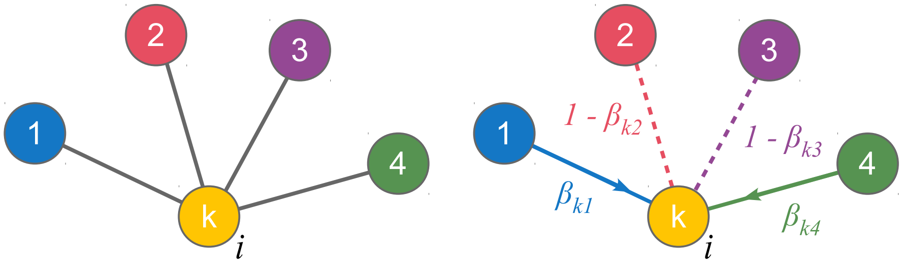

Figure S1. Example of a node with four neighbors, each of which is in a different state. Left panel: each of the neighbors is in a different state, hence the different transmission probabilities. Right panel: Solid colored arrows indicate successful transmissions, while dashed lines indicate an unsuccessful transmission. For node i depicted in the figure, event $e_9 = [1\ \ 0\ \ 0\ \ 1]^T$ has occurred: states 1 and 4 have been successfully transmitted, while states 2 and 3 have not.

and is numerically intractable for nodes with high degree. In order to make the computation of (S2) numerically tractable, in the first part of our approximation we substitute the fractions arising from the number of successful transmissions, $\frac{1}{\sum_{j=1}^{d_i}[e_h]_j}$, with the negative powers of 2, i.e. $\frac{1}{2^{\sum_{j=1}^{d_i}[e_h]_j - 1}}$. For the example on Fig. S1 and for $f^1_{i,k}(t)$ in (S2) this reads

$$\check{f}^1_{i,k}(t) = \beta_{k1}\Big[1 - \frac{1}{2}\beta_{k2} - \frac{1}{2}\beta_{k3} - \frac{1}{2}\beta_{k4} + \frac{1}{4}\beta_{k2}\beta_{k3} + \frac{1}{4}\beta_{k2}\beta_{k4} + \frac{1}{4}\beta_{k3}\beta_{k4} - \frac{1}{8}\beta_{k2}\beta_{k3}\beta_{k4}\Big], \tag{S3}$$

which results in

$$\check{f}^1_{i,k}(t) = \beta_{k1}(1 - \frac{1}{2}\beta_{k2})(1 - \frac{1}{2}\beta_{k3})(1 - \frac{1}{2}\beta_{k4}) = \frac{\beta_{k1}}{1 - \frac{1}{2}\beta_{k1}} \prod_{j=1}^{4}(1 - \frac{1}{2}\beta_{kj}).$$

On the other hand, for the second part of our approximation, note that

$$\sum_{l=1}^{m} f^l_{i,k}(t) + g^0_{i,k}(t) = 1, \tag{S4}$$

i.e. that the probabilities of adopting all possible states from the neighbors together with the probability of adopting none of the states sum to 1. Since $\check{f}^1_{i,k}(t) \neq f^1_{i,k}(t)$ one needs to normalize the approximation in order for it to satisfy (S4). This also ensures that $\sum_{k=1}^{m} p_{i,k}(t) = 1$. Because in the particular configuration of neighboring states each state is present only once, $\check{f}^l_{i,k}(t)$ for $l \in \{1, 2, 3, 4\}$, by analogy with $\check{f}^1_{i,k}(t)$, are

$$\check{f}^l_{i,k}(t) = \frac{\beta_{kl}}{1 - \frac{1}{2}\beta_{kl}} \prod_{j=1}^{4}(1 - \frac{1}{2}\beta_{kj}).$$

Writing Eq. (S4) for the approximate values $\check{f}^l_{i,k}(t)$ gives

$$\sum_{l=1}^{4} \frac{\beta_{kl}}{1 - \frac{1}{2}\beta_{kl}} \prod_{j=1}^{4}(1 - \frac{1}{2}\beta_{kj}) \approx 1 - g^0_{i,k}(t).$$

Here we make use of the product term which is common to all $\check{f}^l_{i,k}(t)$, as a normalizing constant, to normalize the approximation. I.e., by taking

$$\prod_{j=1}^{4}(1-\frac{1}{2}\beta_{kj}) = \frac{1-g^0_{i,k}(t)}{\displaystyle\sum_{l=1}^{4}\frac{\beta_{kl}}{1-\frac{1}{2}\beta_{kl}}}$$

and replacing it in $\check{f}^l_{i,k}$ one obtains the normalized expression for the approximation

$$\tilde{f}^l_{i,k}(t) = \frac{\dfrac{\beta_{kl}}{1-\frac{1}{2}\beta_{kl}}}{\displaystyle\sum_{l=1}^{4}\frac{\beta_{kl}}{1-\frac{1}{2}\beta_{kl}}}(1-g^0_{i,k}(t)).$$

Next, we will derive the same approximation for (5) for the general case. Consider a node i with degree d_i in state k with a particular configuration $c_q = c$ of neighboring states. The difference from the previous case is that the particular configuration is not specifically c_{27}. We calculate the probability $f^{r_x}_{i,k}(t)$ of successful transmission of the state of neighbor x, r_x. Without loss of generality, let $r_x = l$. We first assume that the states of the other neighbors are different from l. Since $s^c_{j,r_j}(t) = 1$ for the specific states r_j present at the d_i neighbors, Eq. (6) reads

$$p(e_h, c_q) = \prod_{j\in N_i}[\beta_{r_ir_j}[e_h]_j + (1-\beta_{r_ir_j})(1-[e_h]_j)].$$

Also, another result of the particular configuration of neighboring states is that the first sum in (5) will be missing. The fact that only neighbor x is considered means that the third sum, the one in the numerator of (5), will also be absent from $f^l_{i,k}(t)$. In this case we have that

$$f^l_{i,k}(t) = \sum_{h=0}^{2^{d_i}-1}\frac{p(e_h)[e_h]_x s_{x,l}(t)}{\sum_{j=1}^{d_i}[e_h]_j}, \tag{S5}$$

where $s_{x,r_x}(t) = s^c_{x,r_x}(t)$. Replacing the fractions $1/\sum_{j=1}^{d_i}[e_h]_j$ by the negative powers of 2 gives

$$\begin{aligned}\check{f}^l_{i,k}(t) &= \sum_{h=0}^{2^{d_i}-1}\frac{p(e_h)[e_h]_x s_{x,l}(t)}{2^{\sum_{j=1}^{d_i}[e_h]_j-1}}\\ &= \sum_{h=0}^{2^{d_i}-1}\frac{2[e_h]_x s_{x,l}(t)}{2^{\sum_{j=1}^{d_i}[e_h]_j}}\prod_{j\in N_i}(\beta_{kr_j}[e_h]_j + (1-\beta_{kr_j})(1-[e_h]_j)).\end{aligned} \tag{S6}$$

If we rewrite the term $1/2^{\sum_{j=1}^{d_i}[e_h]_j}$ as

$$\frac{1}{2^{\sum_{j=1}^{d_i}[e_h]_j}} = \prod_{j\in N_i}(\frac{1}{2}[e_h]_j + (1-[e_h]_j)1) = \prod_{j\in N_i}(1-\frac{1}{2}[e_h]_j), \tag{S7}$$

we have that

$$\begin{aligned}
\check{f}^l_{i,k}(t) &= 2\sum_{h=0}^{2^{d_i}-1}[e_h]_x s_{x,l}(t)\prod_{j\in N_i}(1-\frac{1}{2}[e_h]_j)\prod_{j\in N_i}(\beta_{kr_j}[e_h]_j+(1-\beta_{kr_j})(1-[e_h]_j)) \\
&= 2\sum_{h=0}^{2^{d_i}-1}[e_h]_x s_{x,l}(t)\prod_{j\in N_i}\left(1-\frac{1}{2}[e_h]_j\right)\left(\beta_{kr_j}[e_h]_j+(1-\beta_{kr_j})(1-[e_h]_j)\right) \\
&= 2\sum_{h=0}^{2^{d_i}-1}[e_h]_x s_{x,l}(t)\prod_{j\in N_i}\Big[\beta_{kr_j}[e_h]_j+(1-\beta_{kr_j})(1-[e_h]_j)-\frac{1}{2}\beta_{kr_j}[e_h]_j^2 \\
&-\frac{1}{2}(1-\beta_{kr_j})([e_h]_j-[e_h]_j^2)\Big] \\
&= 2\sum_{h=0}^{2^{d_i}-1}[e_h]_x s_{x,l}(t)\prod_{j\in N_i}[\frac{1}{2}\beta_{kr_j}[e_h]_j+(1-\beta_{kr_j})(1-[e_h]_j))],
\end{aligned} \tag{S8}$$

where for the last equality $[e_h]_j^2=[e_h]_j$ has been used. Further, we separate the term which corresponds to a successful transmission of the state of neighbor x (this means that $[e_h]_x=1$) from the product term in (S8) and obtain

$$\begin{aligned}
\check{f}^l_{i,k}(t) &= 2\sum_{h=0}^{2^{d_i}-1}[e_h]_x s_{x,l}(t)\frac{1}{2}\beta_{kl}\prod_{\substack{j\in N_i,\\ j\neq x}}[\frac{1}{2}\beta_{kr_j}[e_h]_j+(1-\beta_{kr_j})(1-[e_h]_j)] \\
&= \beta_{kl}s_{x,l}(t)\sum_{\substack{e_h\in E_i\\ \setminus\{e_f|[e_f]_x=0\}}}\prod_{\substack{j\in N_i,\\ j\neq x}}[\frac{1}{2}\beta_{kr_j}[e_h]_j+(1-\beta_{kr_j})(1-[e_h]_j)],
\end{aligned} \tag{S9}$$

where $E_i\setminus\{e_f|[e_f]_x=0\}$ is the set of all events e_h where the state transmission from neighbor x is successful, i.e. $[e_h]_x=1$. That is, this set consists of the vectors e_h which are all the binary permutations of size d_i where the x-th component is 1. This allows us to replace the sum in the last row of (S9) with sums over the values of the components of the vectors e_h. These will equivalently go over the same events in the set $E_i\setminus\{e_f|[e_f]_x=0\}$. This is given in (S10), which derives the final expression for the approximation $\check{f}^l_{i,k}(t)$.

$$\begin{aligned}
\check{f}^l_{i,k}(t) &= \beta_{kl}s_{x,l}(t)\sum_{z_1=0}^{1}\sum_{z_2=0}^{1}\cdots\sum_{z_{x-1}=0}^{1}\sum_{z_{x+1}=0}^{1}\cdots\sum_{z_{d_i}=0}^{1}\prod_{\substack{j\in N_i,\\ j\neq x}}[\frac{1}{2}\beta_{kr_j}z_j+(1-\beta_{kr_j})(1-z_j)] \\
&= \beta_{kl}s_{x,l}(t)\prod_{\substack{j\in N_i,\\ j\neq x}}\sum_{z_j=0}^{1}[\frac{1}{2}\beta_{kr_j}z_j+(1-\beta_{kr_j})(1-z_j)] \\
&= \beta_{kl}s_{x,l}(t)\prod_{\substack{j\in N_i,\\ j\neq x}}(1-\frac{1}{2}\beta_{kr_j})=\frac{\beta_{kl}}{1-\frac{1}{2}\beta_{kl}}\prod_{j\in N_i}(1-\frac{1}{2}\beta_{kr_j}) \\
&= \frac{\beta_{kl}a_{ix}s_{x,l}(t)}{1-\frac{1}{2}\beta_{kl}a_{ix}}\prod_{j=1}^{N}(1-\frac{1}{2}\beta_{kr_j}a_{ij})
\end{aligned} \tag{S10}$$

Note that we have used the adjacency matrix to denote the neighbors of node i in the last line of (S10). On a side note, one can expand the previous situation to the case where more than one neighbor is in

the same state as neighbor x. Since one can make the same derivation of (S10) for each of the neighbors in state l, $\check{f}^l_{i,k}(t)$ is simply (S10) summed over all neighbors in state l:

$$\check{f}^l_{i,k}(t) = \sum_{j=1}^{N} a_{ij} s_{j,l}(t) \frac{\beta_{kl}}{1-\frac{1}{2}\beta_{kl}} \prod_{j=1}^{N} (1 - \frac{1}{2}\beta_{kr_j} a_{ij}), \tag{S11}$$

where the third sum in (5) is rewritten as $\sum_{j \in N_i} 1 = \sum_{j=1}^{N} a_{ij}$.

Lastly, we extend to the most general case of acquiring state l from the neighbors considering every possible configuration of neighboring states. Now, in this case the first sum in (5) will be present in the expression. We assume that the state of node i is k and we are interested in the probability of receiving state l, at first when only one neighbor, say x, is in state l, i.e. $r_x = l$. This means that the sum in the numerator (5) will be absent from $\tilde{f}^l_{i,k}(t)$. Starting from (5), and then using (6) and approximating the fractions $1/\sum_{j=1}^{d_i}[e_h]_j$ by the negative powers of two, $1/2^{\sum_{j=1}^{d_i}[e_h]_j - 1}$, one has:

$$\begin{aligned} f^l_{i,k}(t) &= \sum_{q=0}^{m^{d_i}-1} \sum_{h=0}^{2^{d_i}-1} \frac{p(e_h, c_q)[e_h]_x s^{c_q}_{x,l}(t)}{\sum_{j=1}^{d_i}[e_h]_j} \\ &\approx \sum_{q=0}^{m^{d_i}-1} \sum_{h=0}^{2^{d_i}-1} \frac{[e_h]_x s^{c_q}_{x,l}(t)}{2^{\sum_{j=1}^{d_i}[e_h]_j - 1}} \prod_{j \in N_i} \left[\beta_{kr_j}[e_h]_j + (1-\beta_{kr_j})(1-[e_h]_j)\right] s^{c_q}_{j,r_j}(t) \\ &= \sum_{q=0}^{m^{d_i}-1} \sum_{h=0}^{2^{d_i}-1} \frac{2[e_h]_x s^{c_q}_{x,l}(t)}{2^{\sum_{j=1}^{d_i}[e_h]_j}} \prod_{j \in N_i} \left[\beta_{kr_j}[e_h]_j + (1-\beta_{kr_j})(1-[e_h]_j)\right] s^{c_q}_{j,r_j}(t) \end{aligned} \tag{S12}$$

Analogously to the previous case, we replace the term $1/2^{\sum_{j=1}^{d_i}[e_h]_j}$ using (S7) and we obtain that

$$\begin{aligned} \check{f}^l_{i,k}(t) &= 2 \sum_{q=0}^{m^{d_i}-1} \sum_{h=0}^{2^{d_i}-1} [e_h]_x s^{c_q}_{x,l}(t) \prod_{j \in N_i} \left(1 - \frac{1}{2}[e_h]_j\right) \prod_{j \in N_i} \left[\beta_{kr_j}[e_h]_j + (1-\beta_{kr_j})(1-[e_h]_j)\right] s^{c_q}_{j,r_j}(t) \\ &= 2 \sum_{q=0}^{m^{d_i}-1} \sum_{h=0}^{2^{d_i}-1} [e_h]_x s^{c_q}_{x,l}(t) \prod_{j \in N_i} \left(1 - \frac{1}{2}[e_h]_j\right) \left[\beta_{kr_j}[e_h]_j + (1-\beta_{kr_j})(1-[e_h]_j)\right] s^{c_q}_{j,r_j}(t) \\ &= 2 \sum_{q=0}^{m^{d_i}-1} \sum_{h=0}^{2^{d_i}-1} [e_h]_x s^{c_q}_{x,l}(t) \prod_{j \in N_i} \left[\frac{1}{2}\beta_{kr_j}[e_h]_j + (1-\beta_{kr_j})(1-[e_h]_j)\right] s^{c_q}_{j,r_j}(t), \end{aligned} \tag{S13}$$

where, as previously, the fact that $[e_h]_j^2 = [e_h]_j$ has been used. Again, from the product in (S13) we separate the term arising from neighbor x and note that the sum in that case goes over the set of events $E_i \setminus \{e_f | [e_f]_x = 0\}$ where the transmission of x's state is successful, $[e_h]_x = 1$. Again, these events are represented by the vectors e_h of all the binary permutations of size d_i where the x-th component is 1. As in (S10), we replace the sum going over $E_i \setminus \{e_f | [e_f]_x = 0\}$ with the sums which go over the two possible values of the components of the vectors e_h and thus obtain from (S13) an analogous expression to (S10)

with the sum over all the configurations included:

$$\begin{aligned}\check{f}^l_{i,k}(t) &= 2\sum_{q=0}^{m^{d_i}-1}\sum_{h=0}^{2^{d_i}-1}[e_h]_x s^{c_q}_{x,l}(t)\frac{1}{2}\beta_{kl}\prod_{\substack{j\in N_i,\\ j\neq x}}\left[\frac{1}{2}\beta_{kr_j}[e_h]_j+(1-\beta_{kr_j})(1-[e_h]_j)\right]s^{c_q}_{j,r_j}(t)\\
&= \sum_{q=0}^{m^{d_i}-1}\beta_{kl}s^{c_q}_{x,l}(t)\sum_{\substack{e_h\in E_i\\ \setminus\{e_f|[e_f]_x=0\}}}\prod_{\substack{j\in N_i,\\ j\neq x}}\left[\frac{1}{2}\beta_{kr_j}[e_h]_j+(1-\beta_{kr_j})(1-[e_h]_j)\right]s^{c_q}_{j,r_j}(t)\\
&= \sum_{q=0}^{m^{d_i}-1}\beta_{kl}s^{c_q}_{x,l}(t)\sum_{z_0=0}^{1}\cdots\sum_{z_{x-1}=0}^{1}\sum_{z_{x+1}=0}^{1}\cdots\sum_{z_{d_i}}^{1}\prod_{\substack{j\in N_i,\\ j\neq x}}\left[\frac{1}{2}\beta_{kr_j}z_j+(1-\beta_{kr_j})(1-z_j)\right]s^{c_q}_{j,r_j}(t) \qquad \text{(S14)}\\
&= \sum_{q=0}^{m^{d_i}-1}\beta_{kl}s^{c_q}_{x,l}(t)\prod_{\substack{j\in N_i,\\ j\neq x}}\sum_{z_j=0}^{1}\left[\frac{1}{2}\beta_{kr_j}z_j+(1-\beta_{kr_j})(1-z_j)\right]s^{c_q}_{j,r_j}(t)\\
&= \sum_{q=0}^{m^{d_i}-1}\beta_{kl}s^{c_q}_{x,l}(t)\prod_{\substack{j\in N_i,\\ j\neq x}}\left(1-\frac{1}{2}\beta_{kr_j}\right)s^{c_q}_{j,r_j}(t) = \sum_{q=0}^{m^{d_i}-1}\beta_{kl}s_{x,l}(t)\prod_{\substack{j\in N_i,\\ j\neq x}}\left(1-\frac{1}{2}\beta_{kr_j}s^{c_q}_{j,r_j}(t)\right),\end{aligned}$$

where in the last row $s^{c_q}_{j,r_j}(t)=1$ has been used, since node j is indeed in state r_j. Recall that although we go over all possible configurations of states c_q, in essence only those where exactly neighbor x is in state l are considered. Now note that the sum in (S15) which goes over all possible configurations of neighboring states of node i can equivalently be rewritten as

$$\sum_{q=0}^{m^{d_i}-1}1=\prod_{j\in N_i}\sum_{h=1}^{m}s_{j,h}(t).$$

Using this in (S15) and replacing the indexing in $s^{c_q}_{j,r_j}(t)$ with $s_{j,h}(t)$, $s^{c_q}_{x,l}(t)$ with $s_{x,l}(t)$ and β_{kr_j} with

β_{kh} accordingly, we obtain

$$\begin{aligned}
\check{f}^l_{i,k}(t) &= \prod_{j\in N_i}\sum_{h=1}^{m} s_{j,h}(t)\beta_{kl}s_{x,l}(t)\prod_{\substack{j'\in N_i,\\ j'\neq x}}\left(1-\frac{1}{2}\beta_{kh}s_{j',h}(t)\right)\\
&= \beta_{kl}s_{x,l}(t)\prod_{j\in N_i}\sum_{h=1}^{m} s_{j,h}(t)\prod_{\substack{j'\in N_i,\\ j'\neq x}}\left(1-\frac{1}{2}\beta_{kh}s_{j',h}(t)\right)\\
&= \beta_{kl}s^2_{x,l}(t)\prod_{\substack{j\in N_i,\\ j\neq x}}\sum_{h=1}^{m} s_{j,h}(t)\prod_{\substack{j'\in N_i,\\ j'\neq x}}\left(1-\frac{1}{2}\beta_{kh}s_{j',h}(t)\right)\\
&= \beta_{kl}s_{x,l}(t)\prod_{\substack{j\in N_i,\\ j\neq x}}\sum_{h=1}^{m} s_{j,h}(t)\left(1-\frac{1}{2}\beta_{kh}s_{j,h}(t)\right) \qquad \text{(S15)}\\
&= \beta_{kl}s_{x,l}(t)\prod_{\substack{j\in N_i,\\ j\neq x}}\left(\sum_{h=1}^{m} s_{j,h}(t)-\frac{1}{2}\sum_{h=1}^{m}\beta_{kh}s^2_{j,h}(t)\right)\\
&= \beta_{kl}s_{x,l}(t)\prod_{\substack{j\in N_i,\\ j\neq x}}\left(1-\frac{1}{2}\sum_{h=1}^{m}\beta_{kh}s_{j,h}(t)\right)\\
&= \frac{\beta_{kl}s_{x,l}(t)}{1-\frac{1}{2}\sum_{h=1}^{m}\beta_{kh}s_{x,h}(t)}\prod_{j\in N_i}\left(1-\frac{1}{2}\sum_{h=1}^{m}\beta_{kh}s_{j,h}(t)\right).
\end{aligned}$$

Note that in the last row the sum $\sum_{h=1}^{m}\beta_{kh}s_{x,h}(t)$ in the denominator may be readily substituted with $\beta_{kl}s_{x,l}(t)$, however, we will continue to use it for compatibility with the deterministic case where instead of the state vector s_x there is a probability vector p_x. Finally, assume that more than one neighbor may be in state l. The approximate probability of adopting state l from each of those neighbors separately, in every possible configurations of neighboring states, is given with (S15). Taking into account that more than one neighbor may be in state l introduces the sum in the numerator from (5) and thus we obtain

$$\begin{aligned}
\check{f}^l_{i,k}(t) &= \sum_{j\in N_i}\frac{\beta_{kl}s_{j,l}(t)}{1-\frac{1}{2}\sum_{h=1}^{m}\beta_{kh}s_{j,l}(t)}\prod_{j\in N_i}\left(1-\frac{1}{2}\sum_{h=1}^{m}\beta_{kh}s_{j,l}(t)\right)\\
&= \sum_{j=1}^{N}\frac{\beta_{kl}a_{ij}s_{j,l}(t)}{1-\frac{1}{2}\sum_{h=1}^{m}\beta_{kh}a_{ij}s_{j,l}(t)}\prod_{j=1}^{N}\left(1-\frac{1}{2}\sum_{h=1}^{m}\beta_{kh}a_{ij}s_{j,l}(t)\right).
\end{aligned} \qquad \text{(S16)}$$

Note that in the last line of (S16) we have used the adjacency matrix to denote the neighbors of i. Equation (S16) needs to be normalized since it needs to satisfy

$$\sum_{l=1}^{m} f^l_{i,k}(t) + g^0_{i,k}(t) = 1.$$

We follow the same procedure for the normalization as in the example, i.e. we make use of the product

in (S16) as a normalization constant, and obtain that

$$\prod_{j=1}^{N}\left(1-\frac{1}{2}\sum_{h=1}^{m}\beta_{kh}a_{ij}s_{j,l}(t)\right)=\frac{1-g_{i,k}^{0}(t)}{\sum_{j=1}^{N}\frac{\beta_{kl}a_{ij}s_{j,l}(t)}{1-\frac{1}{2}\sum_{h=1}^{m}\beta_{kh}a_{ij}s_{j,l}(t)}}.$$

Using it in (S16), we have the final expression for the approximation $\tilde{f}_{i,k}^{l}(t)$ of the probability $f_{i,k}(t)$ of adopting state l from any combination of neighboring states

$$\tilde{f}_{i,k}^{l}(t)=\frac{\beta_{kl}\sum_{j=1}^{N}\frac{a_{ij}s_{j,l}(t)}{1-\frac{1}{2}a_{ij}\sum_{h=1}^{m}\beta_{kh}s_{j,h}(t)}}{\sum_{j=1}^{N}\frac{a_{ij}\sum_{h=1}^{m}\beta_{kh}s_{j,h}(t)}{1-\frac{1}{2}a_{ij}\sum_{h=1}^{m}\beta_{kh}s_{j,h}(t)}}\left(1-g_{i,k}^{0}(t)\right). \quad \text{(S17)}$$

S2 Assessment of the accuracy of the approximation

Regarding the accuracy of the approximation (S17), we consider two scenarios as follows. In the first scenario we attempt to assess the effect of the number of neighbors on the accuracy of the approximation. We consider node i in state k whose neighbors can be in two possible states, which we call state 1 and state 2. Starting with two neighbors in state 1 and 2 respectively, we increase the number of neighbors in only one of the states. Fig. S2 and Fig. S3 give the results obtained for parameter values $\beta_{k1}=0.05$, $\beta_{k2}=0.25$ and $\beta_{k1}=0.25$, $\beta_{k2}=0.75$, respectively. We add up to 13 nodes, for a total of up to 15 neighbors. A higher number of neighbors results in simulations that last prohibitively long due to the exponential dependence of (5) on the node degree. The results show that increasing the number of neighbors in one of the states lowers the precision of the approximation and that the approximation is more accurate when the state transmission probabilities are smaller. One can expect the error to be larger for nodes of higher degree. In the second scenario, we examine the dependence of the accuracy of the approximation on the transmission probability. We consider node i in state k with four neighbors, three of which are in states whose transmission probabilities, relative to state k, we do not change, and one whose transmission probability is varied from 0 to 1. Results are presented in Fig. S4. We observe that the approximation usually overestimates the actual probabilities for states with high transmission probabilities, and underestimates those of states with low transmission probabilities. This is the case in Fig. S3, as well. Further numerical experiments have shown that the error of the approximation is low when the transmission probabilities are relatively close to each other or when they are much lower than 1 (lower than 0.25 in our experience).

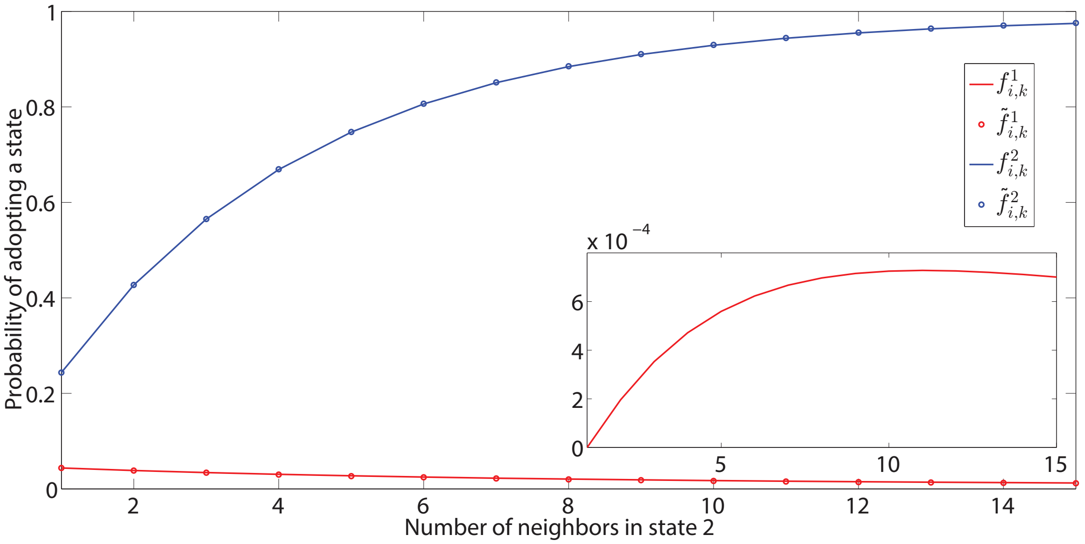

(a) One node is in state 1. Neighbors in state 2 are added.

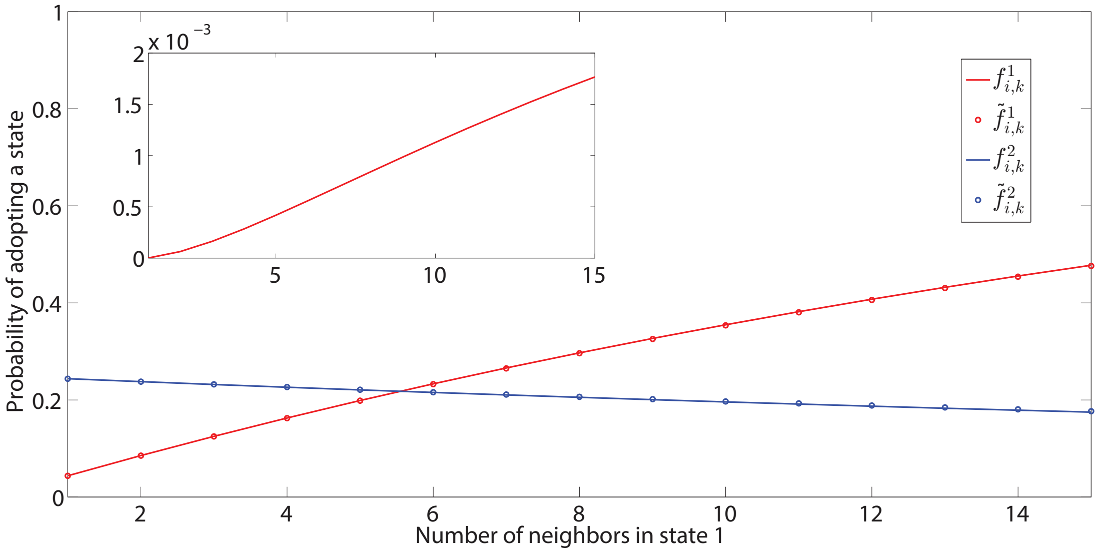

(b) One node is in state 2. Neighbors in state 1 are added.

Figure S2. Assessment of the accuracy of the approximation. Node i is in state k. Its neighbors are in states whose transmission probabilities are $\beta_{k1} = 0.05$ and $\beta_{k2} = 0.25$ relative to state k. Actual and approximated probabilities of adopting a state, as given with (5) and (S17), are presented in the figures with lines and circles, respectively. The inset shows the absolute value of the error of the approximation. Only one curve is shown since the absolute value of the error is equal for both states. The probability of adopting state 1 is underestimated by as much as the probability of adopting state 2 is overestimated.

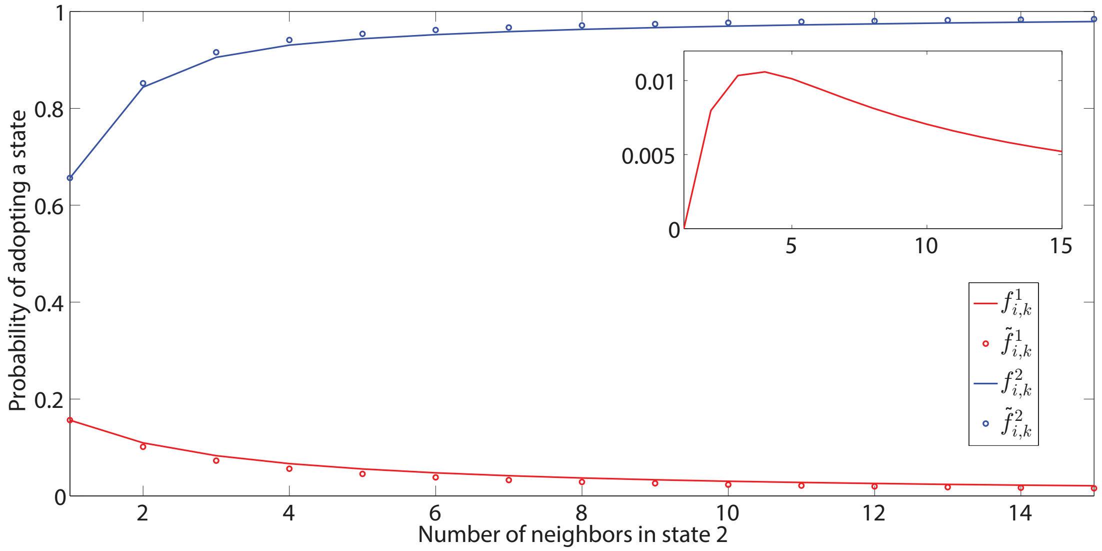

(a) One node is in state 1. Neighbors in state 2 are added.

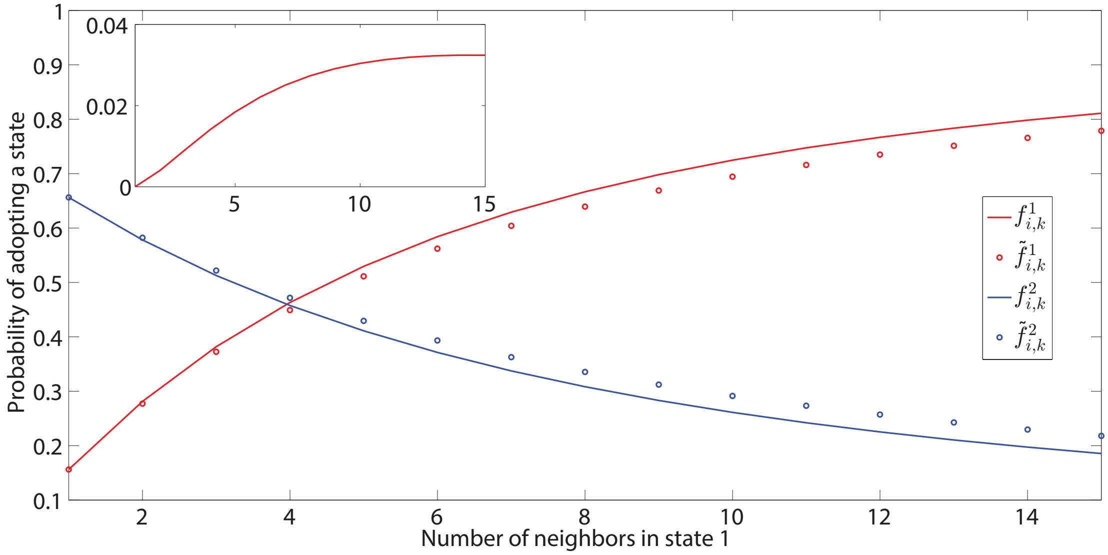

(b) One node is in state 2. Neighbors in state 1 are added.

Figure S3. Assessment of the accuracy of the approximation. Node i is in state k. Its neighbors are in states whose transmission probabilities, relative to state k, are $\beta_{k1} = 0.25$ and $\beta_{k2} = 0.75$. Actual and approximated probabilities of adopting a state, as given with (5) and (S17), are presented in the figures with solid lines and circles, respectively. The inset shows the absolute value of the error of the approximation. Only one curve is shown since the absolute value of the error is equal for both states. The probability of adopting state 1 is underestimated by as much as the probability of adopting state 2 is overestimated.

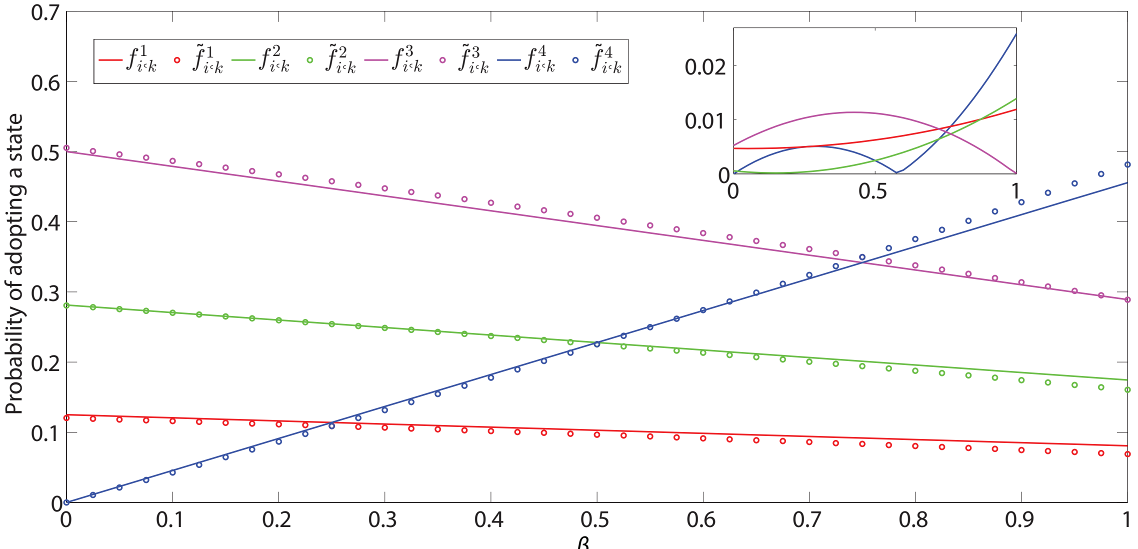

Figure S4. Assessment of the accuracy of the approximation. Neighbors of node i that is in state k have states with transmission probabilities $\beta_{k1} = 0.25$, $\beta_{k2} = 0.5$, $\beta_{k3} = 0.75$, relative to state k. β_{k4} is varied from 0 to 1. Actual and approximated probabilities of adopting a state, as given with (5) and (S17), are presented with solid lines and circles, respectively. The inset shows the absolute value of the error of the approximation for each state.

S3 Derivation of the continuous-time form of the deterministic counterpart of the model

Here we derive the continuous-time form of the deterministic counterpart of our model, presented in Eq. (14) in the main text of the paper. We do this by starting from the discrete-time form of the model, Eqs. (10)–(13) in the main paper, and letting the time step size become infinitesimally small. It is important to distinguish between probability of an event (transmission or spontaneous change), in the discrete-time case, and rate of an event, in the continuous-time case. These two are related by the equation $\hat{\beta}\Delta t = \beta$, where $\hat{\beta}$ is the rate, β is the probability and Δt is the time step size. So, for the differential equation describing the change of the probability that node i is in state l, we have

$$\begin{aligned}
\frac{\mathrm{d}p_{i,l}(t)}{\mathrm{d}t} &= \lim_{\Delta t\to 0} \frac{p_{i,l}(t+\Delta t) - p_{i,l}(t)}{\Delta t} \\
&= \lim_{\Delta t\to 0} \left(\sum_{k=1}^{m} p_{i,k}(t)\hat{\delta}_{kl} + \sum_{k=1}^{m} p_{i,k}(t) \left(\frac{1}{\Delta t} - \sum_{h=1}^{m} \hat{\delta}_{kh} \right) \sum_{h=1}^{m} \tilde{f}^h_{i,k}(t) t^l_{kh} \right. \\
&\quad \left. + \, p_{i,l}(t) \left[\left(\frac{1}{\Delta t} - \sum_{h=1}^{m} \hat{\delta}_{lh} \right) g^0_{i,l}(t) - \frac{1}{\Delta t} \right] \right) \\
&= \sum_{k=1}^{m} p_{i,k}(t)\hat{\delta}_{kl} + \sum_{k=1}^{m} p_{i,k}(t) \sum_{h=1}^{m} t^l_{kh} \lim_{\Delta t\to 0} \frac{\tilde{f}^h_{i,k}(t)}{\Delta t} - \sum_{k=1}^{m} p_{i,k}(t) \sum_{h=1}^{m} \hat{\delta}_{kh} \sum_{h=1}^{m} t^l_{kh} \lim_{\Delta t\to 0} \tilde{f}^h_{i,k}(t) \\
&\quad - p_{i,l}(t) \sum_{h=1}^{m} \hat{\delta}_{lh} \lim_{\Delta t\to 0} g^0_{i,l}(t) - p_{i,l}(t) \lim_{\Delta t\to 0} \frac{1 - g^0_{i,l}(t)}{\Delta t}
\end{aligned} \tag{S18}$$

For brevity, we will omit the argument that describes the current time step in the future derivations. Further, we have

$$\lim_{\Delta t\to 0}\frac{\tilde{f}_{i,k}^{h}}{\Delta t}=\lim_{\Delta t\to 0}\frac{\beta_{kh}\sum_{j=1}^{N}\frac{a_{ij}p_{j,h}}{1-\frac{1}{2}a_{ij}\sum_{v=1}^{m}\beta_{kv}p_{j,v}}}{\Delta t\sum_{j=1}^{N}\frac{a_{ij}\sum_{v=1}^{m}\beta_{kv}p_{j,v}}{1-\frac{1}{2}a_{ij}\sum_{v=1}^{m}\beta_{kv}p_{j,v}}}\left(1-g_{i,k}^{0}\right)$$
$$=\lim_{\Delta t\to 0}\frac{\hat{\beta}_{kh}\sum_{j=1}^{N}\frac{a_{ij}p_{j,h}}{1-\Delta t\frac{1}{2}a_{ij}\sum_{v=1}^{m}\hat{\beta}_{kv}p_{j,v}}}{\Delta t\sum_{j=1}^{N}\frac{a_{ij}\sum_{v=1}^{m}\hat{\beta}_{kv}p_{j,v}}{1-\Delta t\frac{1}{2}a_{ij}\sum_{v=1}^{m}\hat{\beta}_{kv}p_{j,v}}}\left(1-\prod_{j=1}^{N}(1-\Delta t a_{ij}\sum_{v=1}^{m}\hat{\beta}_{kv}p_{j,v})\right) \tag{S19}$$

Here we can apply a general form of the Weierstrass product inequality, $\prod_{j=1}^{N}(1-x_j)\approx 1-\sum_{j=1}^{N}x_j$ where $x_j\ll 1$, $\forall j$, a very commonly used approximation in the epidemic spreading literature. Hence

$$\lim_{\Delta t\to 0}\frac{\tilde{f}_{i,k}^{h}}{\Delta t}\approx\lim_{\Delta t\to 0}\frac{\hat{\beta}_{kh}\sum_{j=1}^{N}\frac{a_{ij}p_{j,h}}{1-\Delta t\frac{1}{2}a_{ij}\sum_{v=1}^{m}\hat{\beta}_{kv}p_{j,v}}}{\Delta t\sum_{j=1}^{N}\frac{a_{ij}\sum_{v=1}^{m}\hat{\beta}_{kv}p_{j,v}}{1-\Delta t\frac{1}{2}a_{ij}\sum_{v=1}^{m}\hat{\beta}_{kv}p_{j,v}}}\Delta t\sum_{j=1}^{N}a_{ij}\sum_{v=1}^{m}\hat{\beta}_{kv}p_{j,v}$$
$$\approx\frac{\hat{\beta}_{kh}\sum_{j=1}^{N}\frac{a_{ij}p_{j,h}}{1-\lim_{\Delta t\to 0}\Delta t\frac{1}{2}a_{ij}\sum_{v=1}^{m}\hat{\beta}_{kv}p_{j,v}}}{\sum_{j=1}^{N}\frac{a_{ij}\sum_{v=1}^{m}\hat{\beta}_{kv}p_{j,v}}{1-\lim_{\Delta t\to 0}\Delta t\frac{1}{2}a_{ij}\sum_{v=1}^{m}\hat{\beta}_{kv}p_{j,v}}}\sum_{j=1}^{N}a_{ij}\sum_{v=1}^{m}\hat{\beta}_{kv}p_{j,v} \tag{S20}$$
$$\approx\frac{\hat{\beta}_{kh}\sum_{j=1}^{N}a_{ij}p_{j,h}}{\sum_{j=1}^{N}a_{ij}\sum_{v=1}^{m}\hat{\beta}_{kv}p_{j,v}}\sum_{j=1}^{N}a_{ij}\sum_{v=1}^{m}\hat{\beta}_{kv}p_{j,v}$$
$$\approx\hat{\beta}_{kh}\sum_{j=1}^{N}a_{ij}p_{j,h}$$

Similarly, we have

$$\lim_{\Delta t\to 0}\tilde{f}_{i,k}^{h}\approx\frac{\hat{\beta}_{kh}\sum_{j=1}^{N}\dfrac{\lim\limits_{\Delta t\to 0}\Delta t a_{ij}p_{j,h}}{1-\lim\limits_{\Delta t\to 0}\Delta t\frac{1}{2}a_{ij}\sum_{v=1}^{m}\hat{\beta}_{kv}p_{j,v}}}{\sum_{j=1}^{N}\dfrac{a_{ij}\sum_{v=1}^{m}\hat{\beta}_{kv}p_{j,v}}{1-\lim\limits_{\Delta t\to 0}\Delta t\frac{1}{2}a_{ij}\sum_{v=1}^{m}\hat{\beta}_{kv}p_{j,v}}}\sum_{j=1}^{N}a_{ij}\sum_{v=1}^{m}\hat{\beta}_{kv}p_{j,v}=0 \tag{S21}$$

$$\lim_{\Delta t\to 0}g_{i,l}^{0}\approx\lim_{\Delta t\to 0}\left(1-\Delta t\sum_{j=1}^{N}a_{ij}\sum_{h=1}^{m}\hat{\beta}_{lh}p_{j,h}\right)=1 \tag{S22}$$

$$\lim_{\Delta t\to 0}\frac{1-g_{i,l}^{0}}{\Delta t}\approx\lim_{\Delta t\to 0}\frac{\Delta t\sum_{j=1}^{N}a_{ij}\sum_{h=1}^{m}\hat{\beta}_{lh}p_{j,h}}{\Delta t}=\sum_{j=1}^{N}a_{ij}\sum_{h=1}^{m}\hat{\beta}_{lh}p_{j,h} \tag{S23}$$

Applying results from Eqs. (S19)–(S23) to Eq. (S18) we obtain the general continuous-time form of the deterministic counterpart of our model

$$\frac{\mathrm{d}p_{i,l}}{\mathrm{d}t}=\sum_{k=1}^{m}p_{i,k}\hat{\delta}_{kl}+\sum_{k=1}^{m}p_{i,k}\sum_{h=1}^{m}\hat{\beta}_{kh}t_{kh}^{l}\sum_{j=1}^{N}a_{ij}p_{j,h}-p_{i,l}\sum_{k=1}^{m}\hat{\delta}_{lk}-p_{i,l}\sum_{j=1}^{N}a_{ij}\sum_{h=1}^{m}\hat{\beta}_{lh}p_{j,h} \tag{S24}$$
